# Control of Multicellular Development by the Physically Interacting Deneddylases DEN1/DenA and COP9 Signalosome

**DOI:** 10.1371/journal.pgen.1003275

**Published:** 2013-02-07

**Authors:** Martin Christmann, Tilo Schmaler, Colin Gordon, Xiaohua Huang, Özgür Bayram, Josua Schinke, Sina Stumpf, Wolfgang Dubiel, Gerhard H. Braus

**Affiliations:** 1 Department of Molecular Microbiology and Genetics, Institute of Microbiology and Genetics, Georg-August-Universität Göttingen, Göttingen, Germany; 2 Department of General, Visceral, Vascular and Thoracic Surgery, Division of Molecular Biology, Charité–Universitätsmedizin Berlin, Berlin, Germany; 3 Medical Research Council, Human Genetics Unit, Western General Hospital, Edinburgh, United Kingdom; Duke University Medical Center, United States of America

## Abstract

Deneddylases remove the ubiquitin-like protein Nedd8 from modified proteins. An increased deneddylase activity has been associated with various human cancers. In contrast, we show here that a mutant strain of the model fungus *Aspergillus nidulans* deficient in two deneddylases is viable but can only grow as a filament and is highly impaired for multicellular development. The DEN1/DenA and the COP9 signalosome (CSN) deneddylases physically interact in *A. nidulans* as well as in human cells, and CSN targets DEN1/DenA for protein degradation. Fungal development responds to light and requires both deneddylases for an appropriate light reaction. In contrast to CSN, which is necessary for sexual development, DEN1/DenA is required for asexual development. The CSN-DEN1/DenA interaction that affects DEN1/DenA protein levels presumably balances cellular deneddylase activity. A deneddylase disequilibrium impairs multicellular development and suggests that control of deneddylase activity is important for multicellular development.

## Author Summary

The family of small ubiquitin-like (Ubl) proteins plays a major role in the control of stability, activity, or localization of modified target proteins in a eukaryotic cell. Lysine side chains are modified by covalent Ubl attachment, and this process can be reversed by specific proteases. Nedd8 is the closest relative to ubiquitin in the Ubl family. We describe here a novel, conserved interplay between two physically interacting deneddylases that are specific for Nedd8. Increased deneddylase activity had been shown to be associated with human cancers. We convey here specific distinct developmental functions of the two deneddylases in multicellular differentiation of the filamentous fungus *Aspergillus nidulans*. The physical interaction between both proteins affects protein stability and therefore cellular deneddylase activity. The equilibrium between the two deneddylases and their physical interaction are conserved from fungi to human and seem to be important for normal development of a multicellular organism. These findings open a different angle for future studies of tumor formation in humans.

## Introduction

Conjugation and deconjugation of target proteins with ubiquitin (Ub) and related proteins is an important posttranslational regulatory principle to control the stability, activity or location of modified substrates. The neuronal precursor cell expressed developmentally down-regulated gene 8 (Nedd8) is a member of the ubiquitin family and represents the closest relative of ubiquitin (Ub) within the group of ubiquitin-like (Ubl) proteins [1]. Covalent attachment of Ubls as Nedd8 requires processing of a precursor peptide resulting in a free di-glycine motif at the C-terminus. All Ubls require in addition an ATP dependent activation cascade of an activating E1, a conjugating E2 enzyme and a substrate specific E3 ligase. Covalent attachment of Nedd8 is termed neddylation, whereas deneddylation is the reverse deconjugation reaction [2]. The major neddylation targets are the cullins which are scaffold proteins within the Cullin-RING ligases (CRL) which serve as ubiquitin ligases. Mammals have seven different cullins (CUL1, 2, 3, 4A, 4B, 5 and 7) [3] while orthologs of three of them (CulA, C and D) are conserved in *Aspergillus nidulans* [4]. Studies in mammalian cells showed that the Ub E3 CRL-RING component Rbx1 interacts with the Nedd8 E2 enzyme Ubc12 and acts as a Nedd8 E3 ligase for cullins [5]. Nedd8 induces a conformational change that allows the first Ub to bridge a gap between the Ub-E2 and the substrate to be ubiquitinated [6]. The CUL1 containing CRLs have more than 350 substrates which include a number of factors involved in human tumor formation [7].

Deneddylation of cullins inactivates CRLs and allows the disassembly of the enzyme complex and the binding of CAND1 (Cullin associated Nedd8 dissociated protein 1) [8], [9]. CAND1 is important for the reassembly of E3 complexes [10]. Thus, deneddylation of cullins has two functions: it blocks ubiquitination and prepares rearrangement of Ub E3 CRLs. The most prominent deneddylases in humans are the CSN (COP9 signalosome) complex and DEN1 (deneddylase 1) [11], [12]. In addition, there are Ub-specific proteases with dual specificity for Ub and Nedd8 such as USP21 and UCH-L3 (Ub-C-terminal hydrolase) [13].

**Figure 1:**
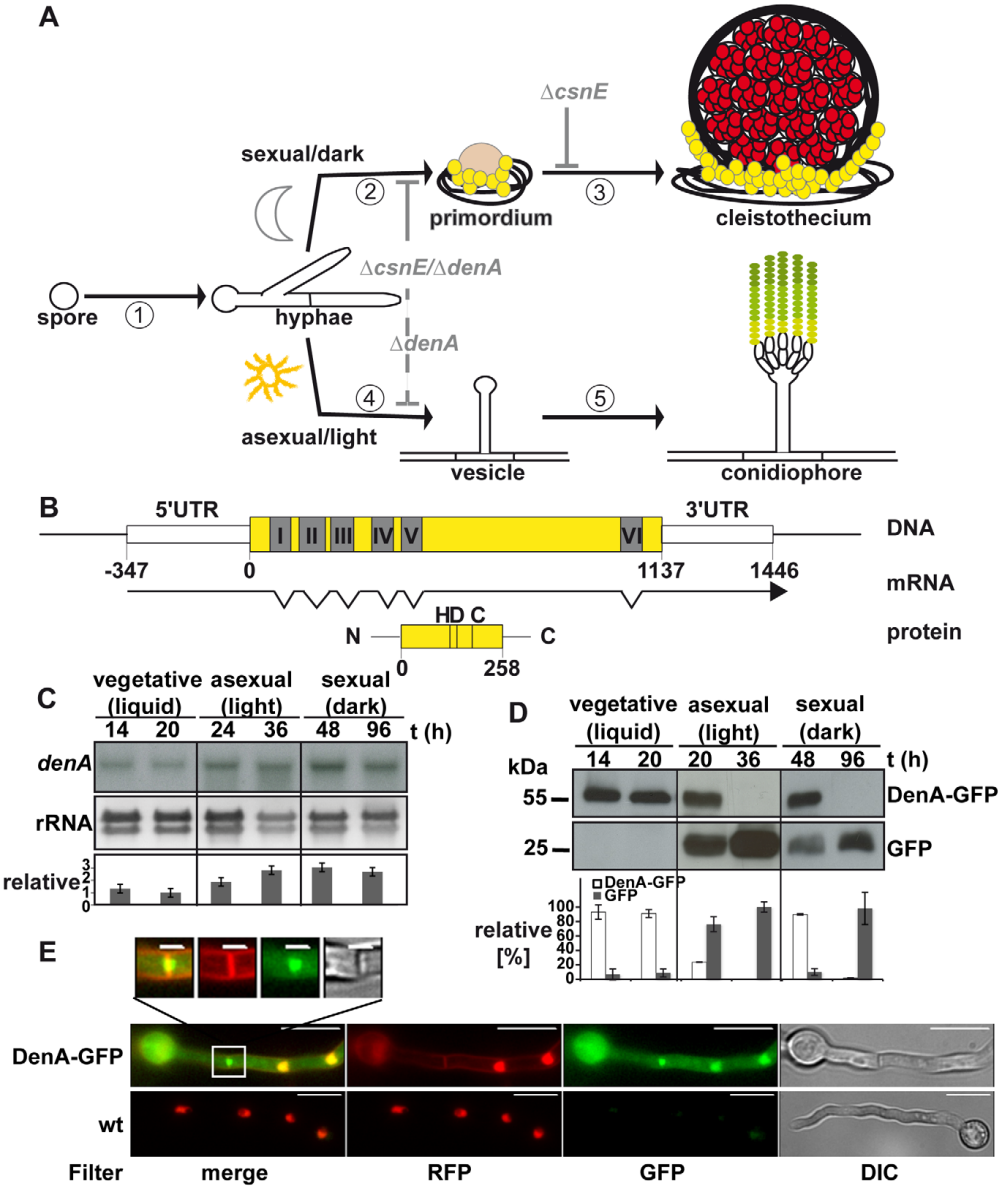
Expression and localization of the DEN1 homolog DenA (AN10456) during development of the fungus *Aspergillus nidulans*. (A) Life cycle of *A. nidulans*. 16 to 20 hours after spore germination vegetative filaments (hyphae) reach developmental competence [1]. In darkness [2] specialized filaments and globular, multi-nuclear cells (Hülle cells; yellow) embed the evolving developmental structure (primordium), which maturates [3] to the sexual fruit body (cleistothecium) within 7 days. In the presence of light [4] an aerial filament with an apical vesicle forms the asexual developmental structure (conidiophore) [5] and releases the uninucleate conidiospores (green) into the air. Arrest points of the described deneddylase mutants are indicated in light grey. Sexual development is blocked (solid grey line) at the stage of primordia in Δ*csnE* and even earlier in the double deletion strain (Δ*csnE*/Δ*denA*). Asexual development is drastically reduced (dashed grey line) in Δ*denA* as well as in Δ*csnE*/Δ*denA*. (B) *denA* gene, transcript (RACE analysis) and protein. Positions within the coding sequence are given relative to the A of the start codon. The histidine (H), aspartate (D) and cysteine (C) typical for the active site of an Ulp1 family protease are indicated at their relative positions. (C) Northern hybridization analysis of *denA* mRNA at indicated stages of development. *denA* mRNA levels of repeated experiments were normalized to ribosomal RNA (rRNA) and presented as x-fold difference relative to 20 hours vegetative growth (graph). (D) Analysis of DenA protein abundance at indicated stages of development (vegetative, asexual, and sexual). GFP was fused C-terminally to DenA (DenA-GFP). Expression was driven by the native *denA* promoter. Strong accumulation of the released GFP tag (GFP) indicated that high amounts of very unstable fusion protein were produced. The proportion of DenA-GFP fusion protein and the remaining GFP-tag was analyzed quantitative and applied as a measure for DenA protein stability. Relative pixel density values are presented as percent of total GFP signal per lane shared between DenA-GFP and the remaining GFP-tag (n = 2). (E) Localization of C-terminal DenA-GFP in *A. nidulans*. The protein resides in the cytoplasm and accumulates in nuclei and around the septum (scale bar = 10 µm). The septal region is enlarged (white square; scale bar = 2 µm). Nuclei are marked by red fluorescence from ectopically integrated mRFP::H2A, membranes are visualized with FM4-64, as well visible through the RFP-filter.

The mammalian CSN consists of eight subunits (CSN1–CSN8) which are conserved in the filamentous fungus *Aspergillus nidulans* [14], [15]. CSN possesses the deneddylase activity as an intrinsic metalloprotease with a JAMM motif localized on CSN5 [16]. CSN forms functional super complexes with CRLs and removes Nedd8 from cullins *via* its metalloprotease [17]–[19]. CSN is more than a deneddylase, since it is associated with kinases [20] and the de-ubiquitinating protein USP15 [21]. In addition, it acts as an assembly platform for Ub E3 CRLs [19], [22]–[24]. In various organisms the CSN is also a key regulator for light dependent cellular processes [25]–[27]. Reduced CSN function results in embryonic lethality in plants [28], insects [29] or mammals [15], and an early block of sexual development in *A. nidulans* [14], [25] (Figure 1A). CSN up-regulation in various cancers suggests a function in human tumor formation [15], [30].

The mammalian DEN1 cysteine protease was initially classified as a SUMO specific protease [31]. Its high preference for Nedd8 is determined by a number of key residues responsible for the architecture of the enzyme [32]. Human DEN1 is a bifunctional enzyme which can process the C-terminus of Nedd8 producing the free di-glycine motif and deconjugates Nedd8 from cullins [33]. The developmental function of DEN1 is unclear. The two DEN1 homologs in the fission yeast *S. pombe*, NEP1 and NEP2 can deneddylate cullins *in vitro*, but no *in vivo* function is known [34]. *Drosophila* DEN1 deneddylates numerous non-cullin proteins which were highly neddylated in corresponding *DEN1* mutants [35]. For example, DEN1 targets the regulator MDM2 for degradation by deneddylation, whereas MDM2 is stabilized by neddylation [36]. The Drosophila *DEN1* mutation suppresses *Nedd8* mutant lethality [35]. Mammalian DEN1 has been shown to be involved in the regulation of apoptosis. Activated caspases can be neddylated by inhibitors of apoptosis (IAPs) leading to a block of caspase activity. DEN1 reactivates caspases by deneddylation [37].

In this study we describe the first developmental phenotypes of a *denA/DEN1* deletion using the multicellular fungus *A. nidulans*. DenA/DEN1 and CSN are required for development and physically interact. CSN targets DEN1/DenA for protein degradation and this mechanism is conserved in *A. nidulans* and human cells.

## Results

### The fungal DEN1 homolog DenA is required for light-dependent development

Mutant strains defective in the DEN1 deneddylase displaying clear phenotypes have not yet been described. *Aspergillus nidulans* represents a multicellular eukaryotic model which grows as a filament, starting from a fungal spore (Figure 1A). After approximately 20 hours of growth this mold is able to respond to external signals to establish an asexual or a sexual developmental pathway for another round of spore formation [38]. Asexual development is promoted by light and results in mitotic spores (conidia) which are released into the air. The sexual pathway is inhibited by light and results in the formation of meiotic spores within closed complex fruiting bodies (cleistothecia) [25], [39]. Defects in the *A. nidulans* COP9 signalosome deneddylase result in mutant strains which are unresponsive to light and blocked in sexual development [14], [25]. This defect was compared to the deletion phenotype of the second deneddylase encoded by the *DEN1* homolog *denA* (AN10456) of *A. nidulans*.

The respective 258 amino acid protein and the corresponding open reading frame were identified performing a BlastP search with the human DEN1 amino acid sequence (UniProt Acc.-No. Q96LD8) on the *A. nidulans* genome [40] applying the NCBI Blast tool (www.ncbi.nlm.nih.gov). The protein is predicted to be a member of the Ulp1 peptidase family with the characteristic catalytic triad histidine (H), aspartate (D) and cysteine (C). Close homologs were as well found in other *Aspergilli*. The human DEN1 protein is 32% identical with *A. nidulans* DenA (Figure S1). Rapid amplification of cDNA ends (RACE) [41] revealed a transcript with seven exons interrupted by six introns with a total length of 1469 base pairs (Figure 1B). Northern hybridization experiments were performed to monitor expression of *denA* during different stages of fungal development. The corresponding mRNA was present throughout all stages of fungal life with elevated levels during asexual and sexual development (Figure 1C). In order to figure out whether DenA protein abundance correlates with gene expression western blot experiments were performed for comparable time points. *A. nidulans* DenA was fused with GFP at the C-terminus (DenA-GFP) and the construct was introduced to the endogenous *denA* locus, expressed from the native *denA* promoter. The corresponding strain was indistinguishable from wild type indicating that the fusion construct can fulfill *denA* function (data not shown). The GFP tag applied for these experiments was stable towards protein degradation in fungal cells, whereas the DenA protein fused to it was not. We analyzed the quantitative relation between the DenA-GFP fusion and the remaining GFP tag and applied this as a measure for protein stability of DenA-GFP (Figure 1D). DenA-GFP was present during vegetative growth and early stages of development, but was no more detected at later stages of development (Figure 1D). During vegetative growth the fungal cell produced low amounts of stable DenA-GFP. During sexual and asexual development high amounts of unstable DenA were produced. This was represented by the increase of signal intensity for the remaining GFP tag which is a stable remnant from degradation of the DenA fusion protein (Figure 1D, GFP). Altogether the data showed that *denA* expression and protein abundance were not correlating. Observations made especially during asexual and sexual development suggest that some kind of post-translational stability control might exist. Additionally we performed fluorescence microscopy with the DenA-GFP which revealed several subpopulations of DenA in vegetative germlings of *A. nidulans*. Those were localized in the nucleus, at either site of the fungal septum within a ball shaped structure or in the cytoplasm (Figure 1E).

**Figure 2:**
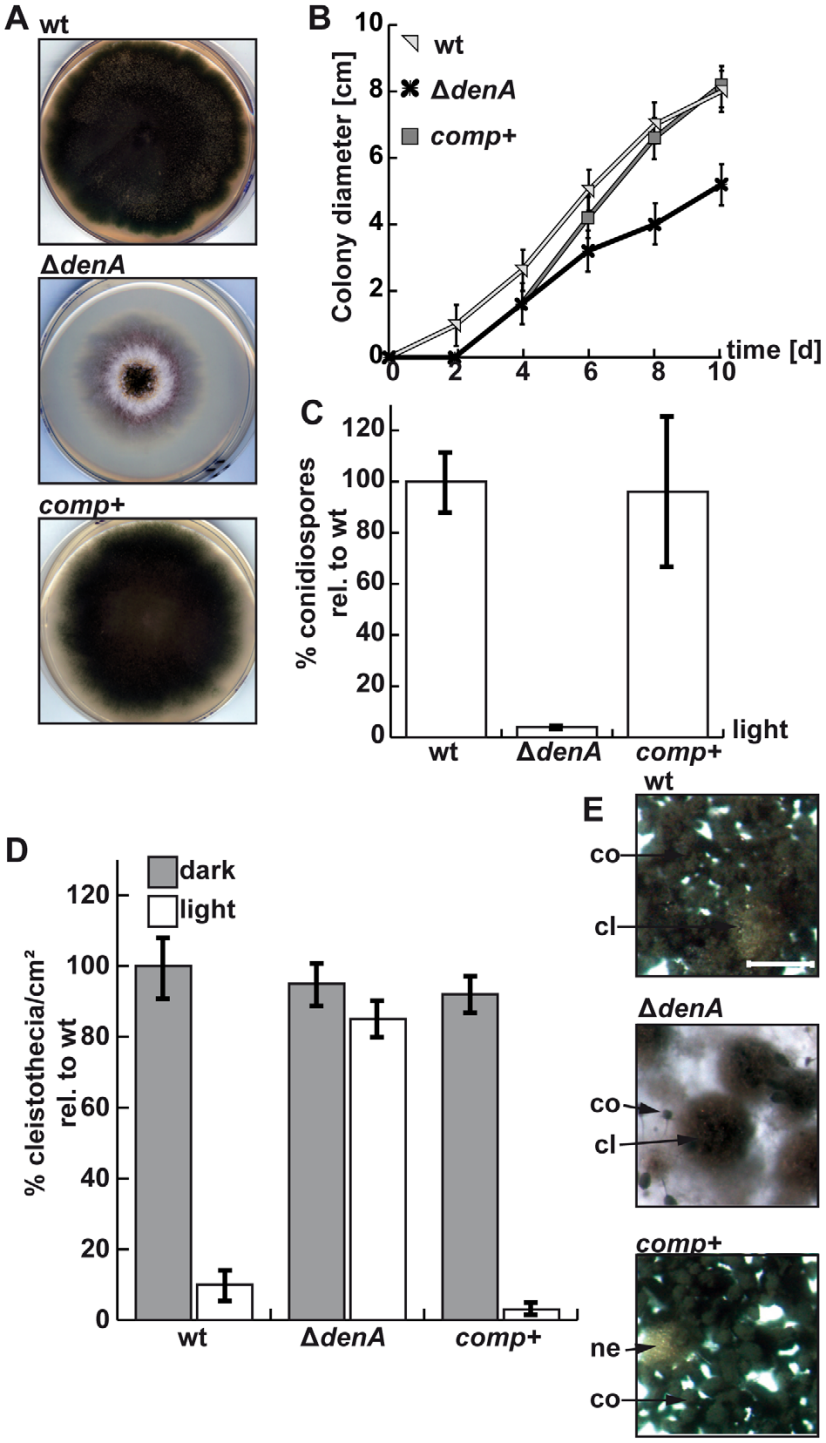
*denA* function is required for *A. nidulans* development. (A) Growth of the *A. nidulans denA* deletion strain. *denA* wild type (wt), *denA* deletion (Δ*denA*) and the complemented deletion strain (*comp+*) were grown on agar plates containing selective minimal medium for 8 days in the presence of light and aeration. (B) Colony diameter of point inoculated colonies monitored over time (d). Plates were incubated at 30°C. (C) Deletion of *denA* causes a 25 fold reduction of spores compared to wild type. Percentage of conidia produced under asexual conditions (30°C, 48 hours, light and normal aeration) compared to wild type (wt). (D) The *A. nidulans denA* deletion strain is impaired in light response of development. Formation of sexual fruit bodies (cleistothecia) of wild type (wt), *denA* deletion (Δ*denA*) and the complemented deletion strain (*comp+*) in light and dark were compared. (E) Agar surface pictures of the corresponding *A. nidulans* strains taken at the 7^th^ day of growth during illumination (asexual conditions) (cl = cleistothecia; co = conidiophores; ne = nest; scale bar = 250 µm).

In order to explore the role of *denA* in the fungal cell we generated a knock-out strain. Deletion of the *denA* coding region resulted in a fungal strain with a significantly reduced growth rate compared to *denA* wild type (Figure 2A, 2B). Complementation of the *ΔdenA* strain by *denA* resulted in a strain indistinguishable from wild type (Figure 2). Asexual development was almost abolished upon *denA* deletion, even during constant white light, which normally favors wild type asexual development. Formation of asexual sporulation structures (conidiophores) took much longer in the Δ*denA* strain and the overall number was marginal. Quantification revealed that spore production corresponds to only 4% of a *denA* wild type strain (Figure 2C). However, the few conidiophores produced by the *denA* mutant differentiated in a normal manner.

There was no significant difference between sexual development of wild type or the *ΔdenA* strain in the dark (Figure 2D, dark columns). However, the *ΔdenA* strain was unresponsive to light and could not inhibit sexual fruit body formation to 20% as wild type in a light-dependent process [42]. The Δ*denA* strain formed similar numbers of cleistothecia with normal size and viable ascospores in light or darkness (Figure 2D, 2E).

Therefore, fungal DenA is important for light-inhibition of sexual development and is required for asexual spore formation (Figure 1A).

### DenA deneddylates cullin *in vivo* and *in vitro*, whereas processed Nedd8 is insufficient to restore the developmental defect of a *denA* deletion

**Figure 3:**
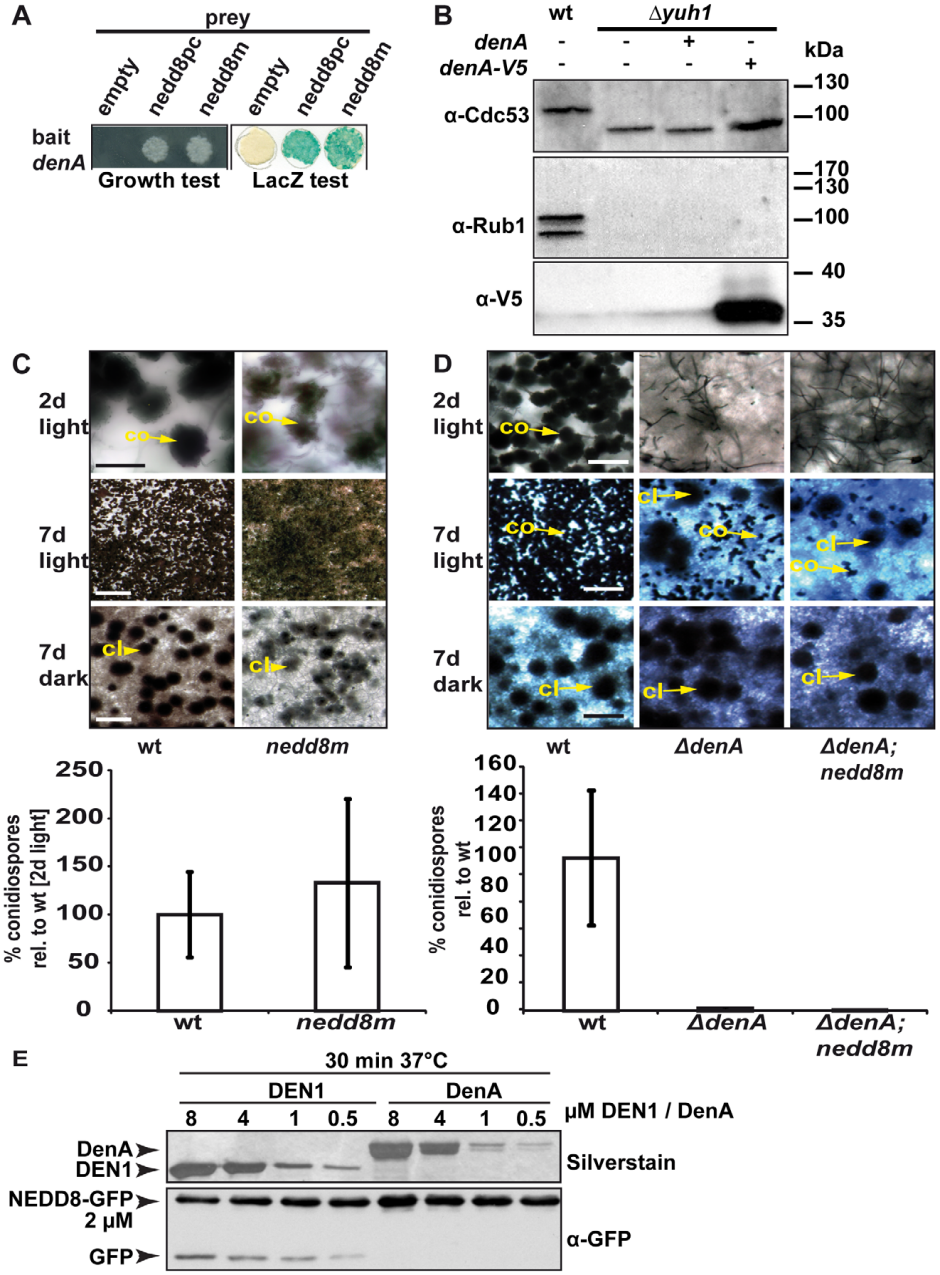
Fungal DenA developmental functions are independent of Nedd8 processing activity. (A) Yeast-2-hybrid interaction between *A. nidulans* DenA and the precursor (nedd8pc) or the mature form (nedd8m) of Nedd8. (B) Western analysis with α-Cdc53 and α-Rub1/Nedd8 to visualize yeast cullin neddylation. Deletion of *yuh1* prevents cleavage of the Rub1 precursor, therefore neddylated protein species can neither be recognized with α-Cdc53 nor with α-Rub1/Nedd8. DenA expression driven by the inducible *GAL1* promoter was applied to test the processing ability of the protease towards the Rub1 precursor. Expression of DenA was visualized with α-V5. DenA expression was not sufficient to restore Rub1 processing in a *yuh1* deficient *S. cerevisiae* strain [44]. (C) An *A. nidulans* strain expressing a mature *nedd8* construct (*nedd8m*) instead of normal *nedd8* (wt) displayed a wild type like phenotype. Asexual structures (co) displayed on agar surface after 2 days of growth in the presence of light (scale bar = 20 µm) and after 7 days (scale bar = 500 µm). Sexual structures (cl) were only formed in the dark, but not in the light after 7 days incubation at 37°C (scale bar = 500 µm). Quantification of asexual spores from both strains after 2 days of incubation at 37°C in light. (D) The *ΔdenA/nedd8m* strain (mature Nedd8) and the *ΔdenA* (precursor Nedd8) are unresponsive to light-dependent inhibition of sexual development (compare rows 2 and 3; scale bar = 50 µm, first row; scale bar = 225 µm, second and third row) and impaired in asexual development (conidiation). (E) Human DEN1 cleaved human Nedd8 C-terminally fused with GFP while *A. nidulans* DenA did not. Nedd8-GFP substrate was combined with decreasing amounts of recombinant DEN1 or DenA, respectively (8–0.5 µM). The reaction mixture was incubated for 30 min at 37°C, immediately denatured, separated by SDS-PAGE and subjected to western blot analysis. α-GFP was applied to monitor cleavage of the Nedd8-GFP substrate. Samples were separated by additional SDS-PAGE and silver stained to prove for the presence of the respective deneddylase.

Human DEN1 is a dual functional protease *in vitro*, processing Nedd8 or cleaving the isopeptide bond between Nedd8 and a target protein [31], [33], [43]. Yeast-2-hybrid experiments revealed that *A. nidulans* DenA interacts with the precursor or the mature form of fungal Nedd8 (Figure 3A). We addressed if processing or deneddylation activity of DenA is responsible for the developmental phenotype. *Saccharomyces cerevisiae* strains deleted for the UCH encoding gene *yuh1* cannot produce the processed variant of yeast Nedd8 (Rub1). Such strains show no obvious growth phenotype but in western experiments neddylated cullins cannot be detected with appropriate antibodies (Figure 3, lane 2) [44]. We introduced a plasmid into the corresponding yeast deletion strain expressing *A. nidulans denA* from an inducible *GAL1* promoter. After induction by growth on galactose containing medium we prepared crude extracts and performed western blot experiments to monitor protein neddylation. Neither the yeast cullin Cdc53 appeared in its neddylated form nor did the Rub1/Nedd8 antibody detect any other neddylated protein at the size of a cullin (Figure 3B). Thus, *A. nidulans* DenA was unable to complement the Nedd8 processing deficient *yuh1* mutant of *S. cerevisiae*. To further discriminate between Nedd8 processing and deneddylation an *A. nidulans* strain was constructed expressing a mature Nedd8 variant (Nedd8m) that does not require processing. The resulting strain was viable and competent for asexual and sexual development. Therefore processed Nedd8 can fulfill the functions of the original unprocessed Nedd8 (Figure 3C) [4]. When the processed Nedd8 mutant was combined with Δ*denA*, it was indistinguishable from the *denA* deletion phenotype (Figure 3D). Furthermore, *in vitro* activity tests with recombinant *A. nidulans* DenA and human DEN1 (Figure 3E and Figure S2) were performed. A linear substrate composed of processed human Nedd8, C-terminally fused with GFP was efficiently cleaved by the human DEN1 protease but the fungal DenA showed no detectable formation of cleavage product under the tested conditions (Figure 3E). These experiments support that the fungal *denA* deletion phenotype, which impairs *A. nidulans* development, does not depend on the Nedd8 processing function.

**Figure 4:**
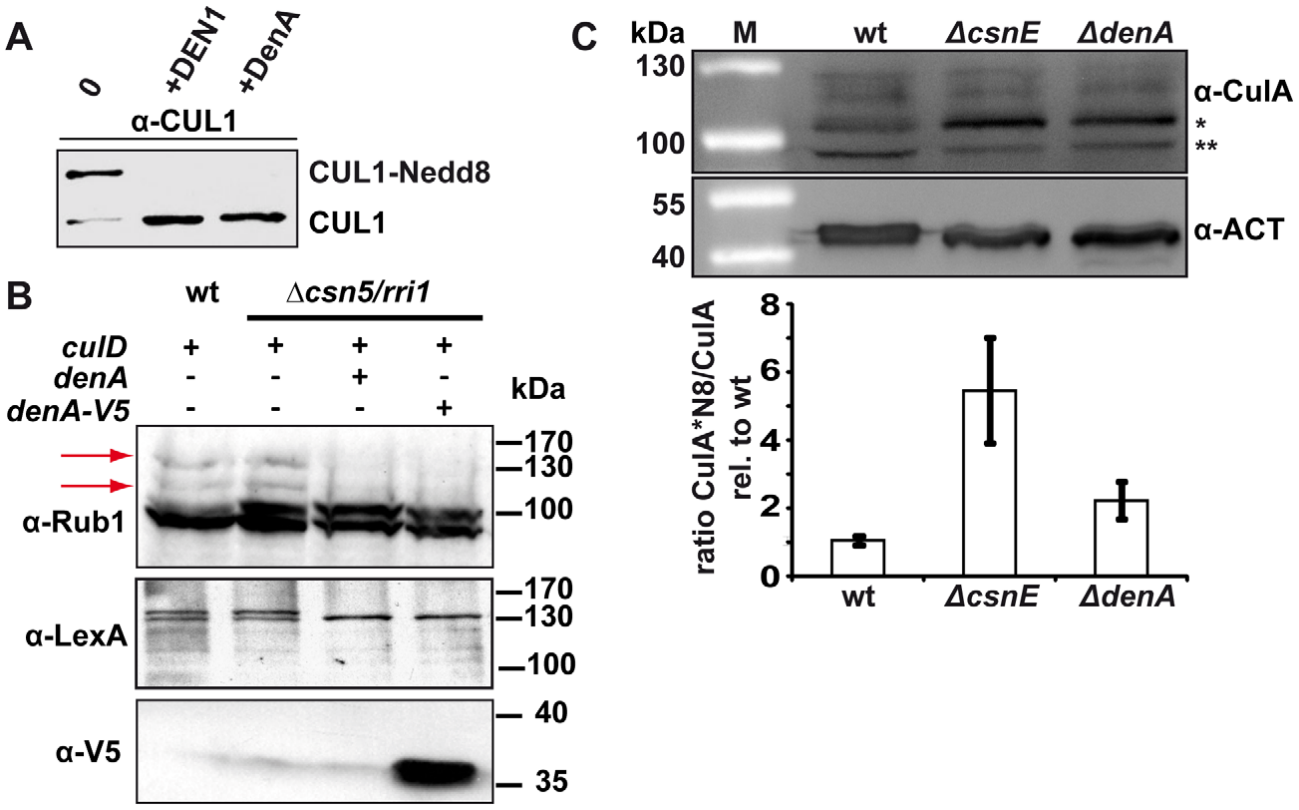
DenA deneddylase activity. (A) Recombinant human DEN1 and fungal DenA deneddylate a human CUL1-Nedd8 substrate *in vitro*. SDS-PAGE and subsequent western analysis show cleavage of the substrate (∼60 kDa) producing the C-terminal CUL1 (∼50 kDa) as outlined in experimental procedures. (B) Deneddylation test in a heterologous yeast system. *A. nidulans* DenA can remove Rub1 from CulD in heterologous expression experiments in *S. cerevisiae*. DenA was expressed as native protein or C-terminally fused with a V5/His6 epitope tag. Both variants were driven by the inducible *GAL1* promoter. CulD, N-terminal-fused with the LexA activation domain, was expressed under control of the constitutive *ADH* promoter. *A. nidulans* proteins were expressed in *S. cerevisiae* wild type and Δ*csn5* background. Western analysis with antibodies against Rub1 (α-Rub1), the LexA epitope (α-LexA) and the V5 epitope (α-V5) were performed. Detection with α-Rub1 generated two additional signals upon *culD* expression, representing LexA-CulD and a second CulD pool where LexA was unspecifically cleaved off. Both signals disappeared upon co-expression of DenA indicating deneddylation activity (red arrows). The slower migrating signal of α-LexA western experiments corresponded to Rub1 modified LexA-CulD. This signal was absent when DenA was co-expressed. Detection of the V5 tag was applied to monitor DenA expression. The neddylated yeast cullin migrating at around 100 kDa was not affected by DenA activity. (C) Deneddylation of fungal CulA by CSN and DenA. Whole cell lysates of *A. nidulans* wild type, Δ*csnE* and Δ*denA* were probed with α-CulA. The ratio of neddylated CulA (CulA*N8; ∼106 kDa*) to non-neddylated CulA (∼96 kDa**) was calculated from three independent experiments. Membranes were reprobed with α-Actin (α-ACT) for normalization.

Deneddylation activity of fungal DenA was examined in more detail. GST-DenA purified from *E. coli* cleaved the isopeptide bond of human CUL1-Nedd8 as efficient as human DEN1 (Figure 4A and Figure S3) demonstrating deneddylase function of the fungal protein. To test DenA deneddylation *in vivo* we accomplished heterologous expression studies in *S. cerevisiae*. A yeast strain constitutively expressing *A. nidulans culD*, the fungal homologue of human *CUL4*, was additionally transformed with a plasmid containing a galactose inducible construct of *A. nidulans denA*. Following induction of *denA* expression by growth on galactose containing medium CulD neddylation was monitored by western experiments (Figure 4B). When DenA was present CulD could not be detected with the Rub1/Nedd8 antibody and also a LexA antibody generated only a signal for the non neddylated form of CulD (Figure 4B, right lanes). Furthermore, whole cell extracts of *A. nidulans* wild type, Δ*csnE* and Δ*denA* were probed with a CulA specific antibody to examine the neddylation state of the cullin. Upon *denA* deletion the portion of neddylated CulA increased compared to the wild type, corroborating DenA function in cullin deneddylation *in vivo*. Neddylated CulA species were increased in strains defective in DenA or in the COP9 signalosome deneddylase CsnE. However, the accumulation of neddylated cullin was less pronounced in Δ*denA* (Figure 4C) indicating that CulA might not be the predominant target of DenA. Nevertheless, these data further support that DenA function in *A. nidulans* development is rather due to deneddylation than to a Nedd8 processing activity.

### Double deletion of *csnE* and *denA* abolishes fungal development and accumulates neddylated proteins

**Figure 5:**
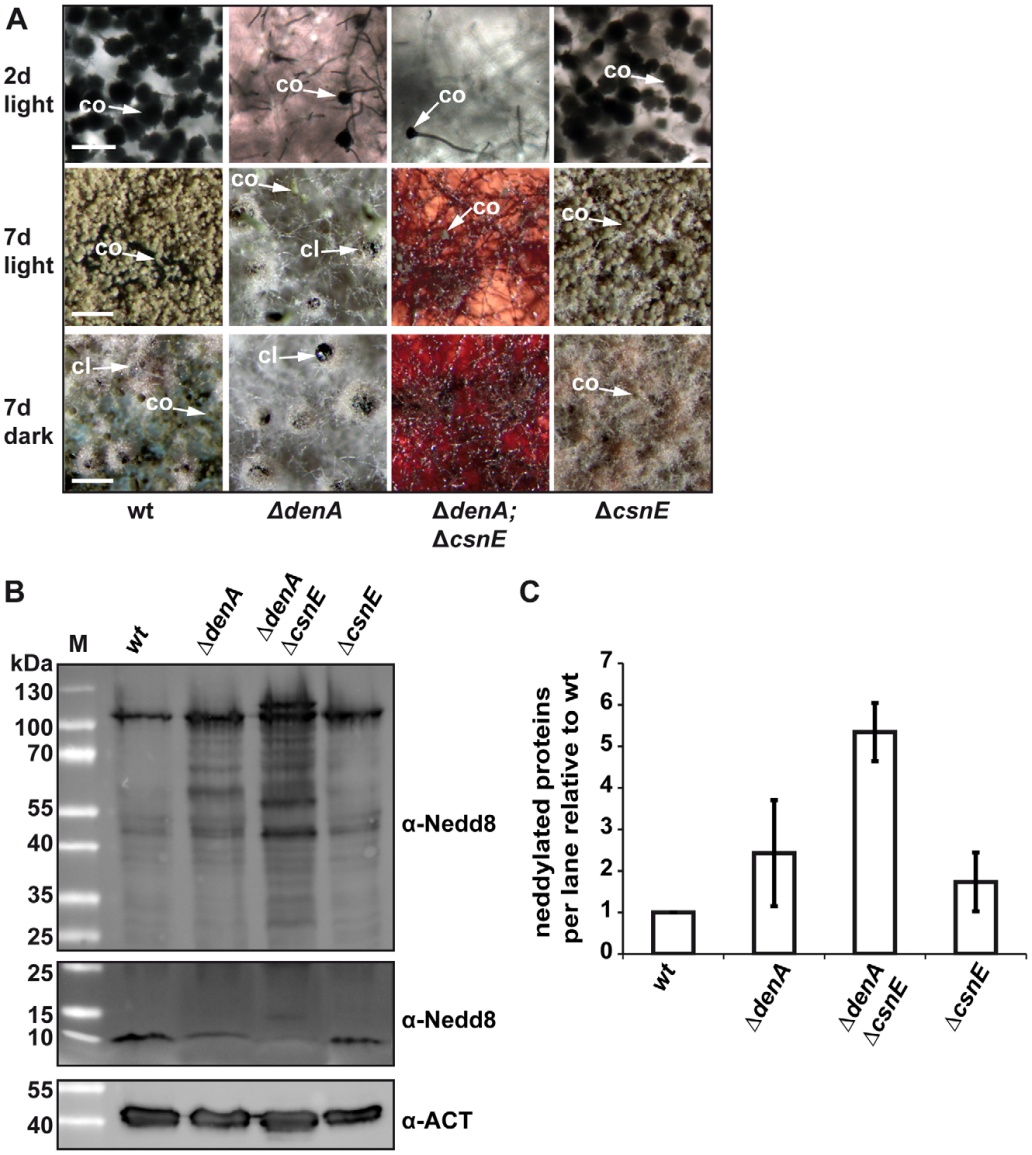
Double knock-out of the deneddylase encoding genes *denA* and *csnE* abolished fungal development. (A) *ΔdenA* and *ΔdenA*/*ΔcsnE* strains were impaired in conidiophore formation while Δ*csnE* and wild type (wt) form similar amounts of asexual structures. Even after seven days of asexual development only marginal numbers of conidiophores are formed in Δ*denA* or Δ*denA*/Δ*csnE* strain. Sexual development after 7 days in the dark occurred for *ΔdenA* but was abolished in *ΔdenA*/*ΔcsnE*. Accumulated red color within the hyphae reflects impaired secondary metabolism. Initiation of sexual development is independent of light in both single deletion strains (7 days, light). Δ*csnE* showed nest (yellowish filaments) formation in light and darkness, but could not progress further in sexual fruit body formation. The red color typical for *csnE* deletion is covered by the layer of hyphae and conidiophores in the pictures displayed here. The *ΔdenA*/*ΔcsnE* was impaired in sexual development and secondary metabolism (scale bar = 50 µm, first row; scale bar = 200 µm, second and third row). (B) Deneddylation deficient *A. nidulans* mutants accumulate neddylated proteins. Western analysis of *A. nidulans* wild type (wt), *ΔdenA*, *ΔcsnE* single deletion strains and *ΔdenA/ΔcsnE* double knock-out strain. Crude extracts were probed with *A. nidulans* α-Nedd8. Gels with identical samples were run for different times to allow proper separation of high migrating signals (higher panel) of neddylated proteins and to preserve the signal of free Nedd8 (lower panel). An increase of neddylated proteins correlates with a reduction of free Nedd8. (C) Semi-quantitive analysis of the Nedd8 signal intensity within each lane for *ΔdenA*, *ΔcsnE* single deletion strains and *ΔdenA/ΔcsnE* double knock-out strain relative to wild type. Signals were normalized by reprobing the membranes with α-Actin (α-ACT). Three independently repeated experiments were included into the quantification.

An *A. nidulans csnE/CSN5* deletion strain defective in the deneddylase subunit of CSN is unresponsive to light and blocked in sexual development. This is accompanied by an altered secondary metabolism represented by an aberrant red color accumulating within the hyphae and the surrounding medium [14], [45], [46]. The *denA* deletion strain was also unresponsive to light and impaired in asexual development. The developmental interplay between the two fungal deneddylases was analyzed by constructing a double mutant. The Δ*denA/ΔcsnE* strain grew poorly but was viable. It combined the phenotypes of both single deletions including the red color phenotype observed in a Δ*csnE* strain (Figure 5A) [14]. The *A. nidulans* double deletion strain was able to grow as vegetative filament. Asexual development was reduced to the marginal level observed in the Δ*denA* strain and sexual development was completely abolished (Figure 5A). This corresponds to Δ*denA* for asexual development, but goes even further than Δ*csnE* which can still initiate but no more complete the sexual cycle [14], [25], [45] (Figure 1A). Western experiments revealed that fungal Nedd8 was detected in crude extracts of *A. nidulans* wild type, Δ*denA*, Δ*csnE* and Δ*denA*/Δ*csnE*. Deletion of either deneddylase alone already resulted in a recognizable increase of the Nedd8 signal compared to wild type. In the Δ*denA* strain this was mainly caused by an intensification of multiple Nedd8 signals across a wide molecular weight range. The Δ*csnE* strain showed a major increase of the Nedd8 signal around 100 kDa which corresponds to the size of neddylated *A. nidulans* cullins. The Δ*denA*/Δ*csnE* strain showed a drastic increase of neddylated proteins, not only compared to wild type, but also to the single deletion strains. This is due to an addition of the effects observed for each single deletion strain and accumulation of an additional band (∼116 kDa) in the Δ*denA/ΔcsnE* strain, which might be due to multiple neddylation of a cullin (Figure 5B–5C). This correlated with reduction of free Nedd8 compared to wild type which was most pronounced in the Δ*denA*/Δ*csnE* strain and to a lower extend also visible in the *denA* and *csnE* single deletion mutants (Figure 5B).

The data suggest that *A. nidulans* without the deneddylases DenA and CSN has lost almost all developmental competence. The double deletion phenotype of only sporadic asexual sporulation and no sexual development is accompanied by an intensive accumulation of neddylated proteins. This suggests that deneddylation by DenA and CSN plays a prominent role in the regulation of fungal developmental programs beyond filamentous growth.

### CSN interacts with DEN1/DenA in fungi and humans

**Figure 6:**
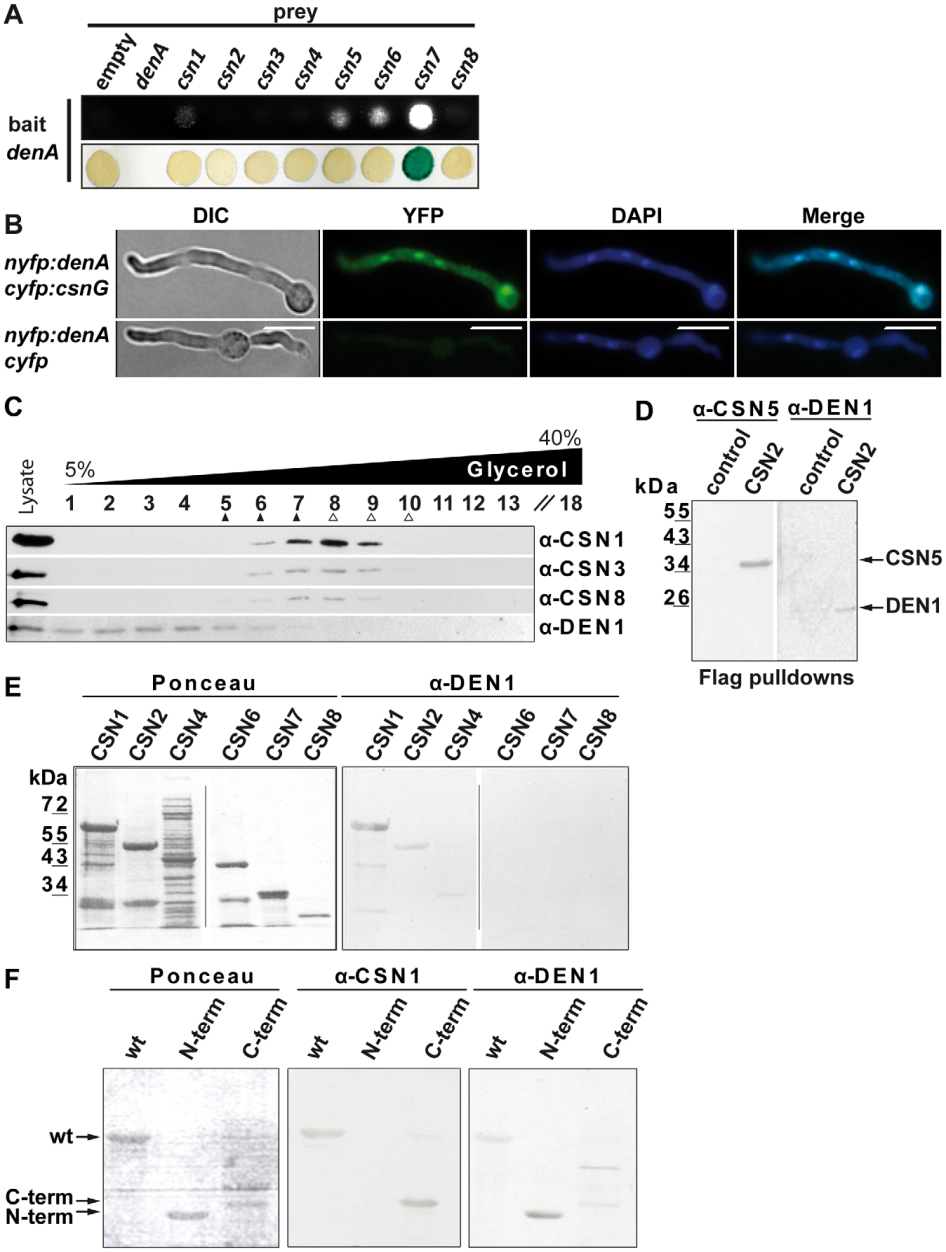
DEN1/DenA deneddylase interacts with the COP9 signalosome (CSN) in fungal and human cells. (A) *A. nidulans* DenA-CSN interactions in yeast-2-hybrid growth and β-galactosidase activity test. (B) DenA and CSN7/CsnG interaction is enriched in the nucleus. BiFC studies in *A. nidulans* for DenA (*nYFP::denA*) and CSN7/CsnG (*cYFP::csnG*) interaction. The control strain expressed only the DenA fusion protein (*nYFP::denA*) whereas *cYFP* was expressed without a fused protein. Nuclei were visualized by DAPI staining (scale bar = 10 µm). (C) Human DEN1-CSN interaction. Density gradient centrifugation of lysate from HeLa cells and subsequent western experiments with fractions of different density. Endogenous DEN1 was detected by the α-DEN1 antibody and the position of the CSN was estimated by α-CSN1, α-CSN3 and α-CSN8 antibodies. The density gradient was calibrated with purified CSN about 400 kDa, ▴) and with purified 20S proteasome (about 700 kDa, Δ). (D) Flag-pull downs from lysates of Flag-CSN2 B8 cells. Proteins specifically eluted with the Flag peptide were analyzed by western experiments. CSN5 and DEN1 were detected by specific antibodies. Control pull downs were performed with lysates from B8 cells. (E) Far-western experiments with recombinant subunits CSN1, CSN2, CSN4, CSN6, CSN7 and CSN8. Membranes were incubated with 1 µg/ml DEN1 protein, washed and probed with the α-DEN1 antibody. (F) Recombinant full-length CSN1 (wt), CSN1(1–221) (N-term) and CSN1(222–527) (C-term) were analyzed by far-western blot. Membranes were incubated with DEN1 as in (E) and probed with the α-CSN1 or the α-DEN1 antibody.

We investigated whether the DenA and CSN deneddylases physically interact within the fungal cell. Yeast-2-hybrid analysis revealed a strong interaction of *A. nidulans* DenA with CsnG/CSN7 and weaker interactions with CsnA/CSN1, CsnE/CSN5 and CsnF/CSN6 of *A. nidulans* (Figure 6A). This suggests an interaction of DenA with the CSN complex. Bi-molecular fluorescence complementation (BiFC) studies [9] were conducted to verify the major interaction of DenA with the CsnG/CSN7 subunit. DenA and CsnG/CSN7 were fused to one half of a split yellow fluorescent protein (YFP) at their N-termini. Expression of the fusion proteins resulted in a fluorescence signal accumulating in the nucleus (Figure 6B) confirming the yeast-2-hybrid result.

An interaction between the DenA deneddylase and the COP9 signalosome deneddylase had not yet been described. We investigated whether DEN1 and CSN also interact in human cells. Separation of HeLa cell lysates by density gradient centrifugation and subsequent western analysis revealed co-sedimentation of CSN and a portion of DEN1 (Figure 6C). This corroborates a physical interaction between the two deneddylases in eukaryotic cells. Flag-pull downs with Flag-CSN2-B8 cells verified the interaction. Flag-labeled CSN complex was pulled down and analyzed by western experiments. These pull downs contain all CSN subunits as well as additional associated proteins [18], [22]. Our analysis demonstrated that DEN1 was co-precipitated with the CSN (Figure 6D).

In order to figure out which CSN subunit mediates the interaction with DEN1 in human cells we performed far western experiments. Selected CSN subunits as shown in Figure 6E were produced recombinant, separated by SDS-PAGE and blotted onto a nitrocellulose membrane. Immuno-blotting with the α-DEN1 antibody showed a strong signal with CSN1 and a weak interaction with CSN2 whereas the other CSN subunits included in the test showed no interaction (Figure 6E, right panel). In control experiments the membranes were stripped and probed again with the α-DEN1 antibody demonstrating that the detected interaction was specific. Additional far-western experiments were performed to specify the site of interaction in CSN1.Wild type human His-CSN1 (1–527), an N-terminal fragment of His-CSN1(1–221) and a C-terminal fragment of His-CSN1(222–527) [22] were applied to these experiments. The results demonstrate a specific binding of DEN1 to His-CSN1wt, as well as to the N-terminal His-CSN1 (1–221) fragment (Figure 6F). All these data indicate a physical interaction between DEN1/DenA and CSN which is conserved from fungi to man.

### CSN targets DEN1/DenA for degradation in fungal and human cells

**Figure 7:**
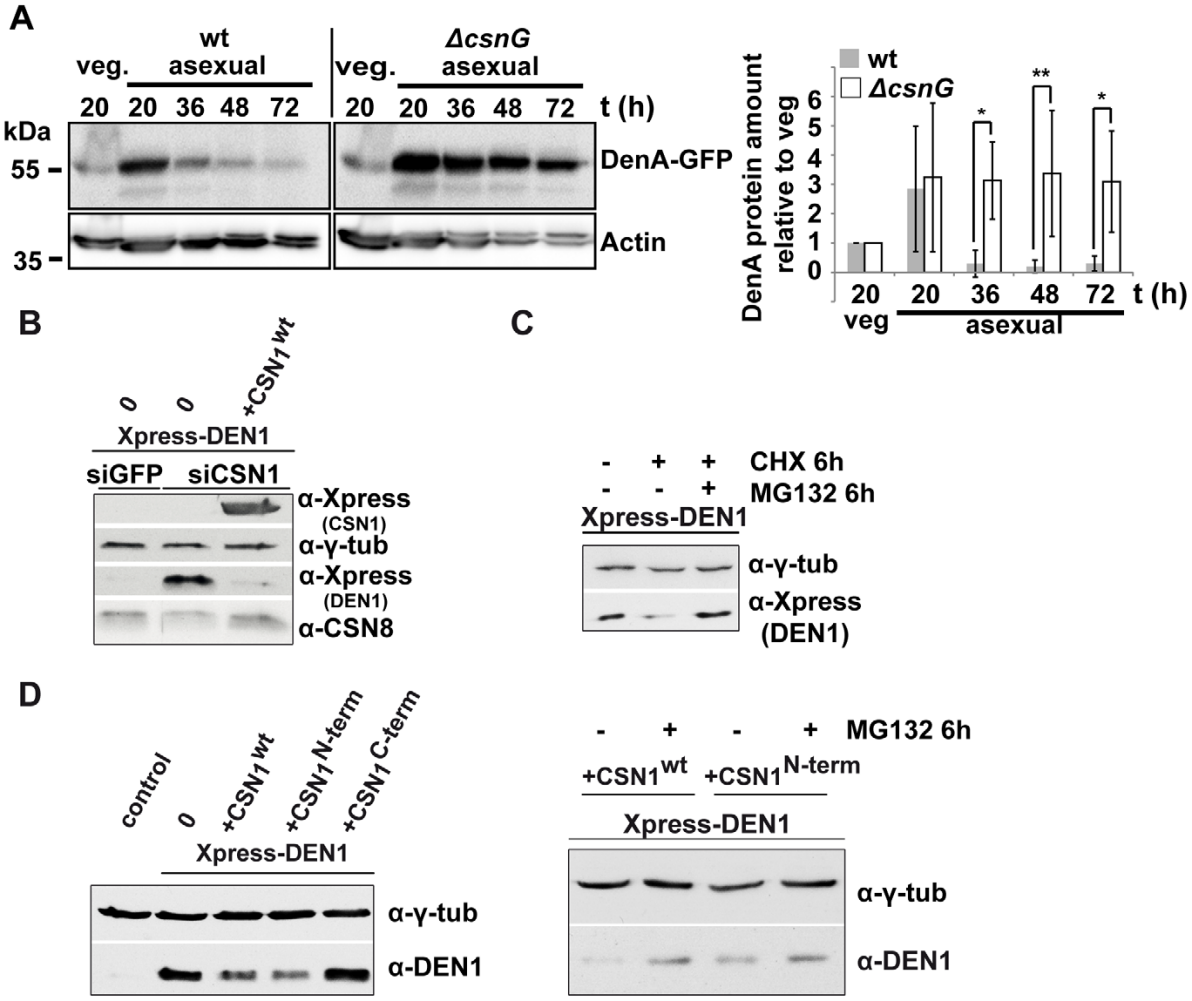
CSN targets DEN1/DenA for degradation in fungal and human cells. (A) Quantitative analysis of repeated western blot experiments displayed the differences in DenA abundance in fungal wild type and *ΔcsnG* cells. DenA levels of the different asexual developmental time points are shown relative to the vegetative (veg.) control for each strain. Anti-Actin was applied as loading control (statistics: 2-way ANOVA; n = 3; *p<0,05, **p<0,01). (B) Xpress-DEN1 was overexpressed in siGFP and siCSN1 human cells and steady state Xpress-DEN1 levels were estimated by western analysis with the α-Xpress antibody. Xpress-CSN1 was overexpressed in siCSN1 cells and DEN1 and CSN8 were probed with appropriate antibodies. (C) Xpress-DEN1 was overexpressed in siGFP human cells and the proteasome inhibitor MG132 was added 6 h before cell lysis at a final concentration of 10 µM. Cyclohexamide (CHX) was added in a final concentration of 10 µg/ml (D) Xpress-DEN1 was co-expressed in HeLa cells together with Xpress-CSN1wt, Xpress-CSN1(1–221) or Xpress-CSN1(222–527) in the absence or in the presence of MG132 (right panel), which was added 6 h before cell lysis. Cells were lyzed 24 h after co-transfection and lysates were analyzed by western blot using the α-DEN1 antibody (0 = only Xpress-DEN1).

The functional impact of the CSN-DEN1/DenA interaction on the molecular level in fungal and human cells was determined. DenA protein levels were monitored in *csn* deficient *A. nidulans* strains during development and compared to wild type cells. Initial experiments revealed that wild type cells produced low amounts of stable DenA protein during vegetative growth but high levels of very unstable DenA during development (Figure 1D; unpublished data). This effect is most prominent during asexual development. We performed repeated western experiments to quantify the effect. Upon deletion of *csnG*, the DenA interacting CSN subunit, DenA-GFP protein stability occurred to be significantly increased along asexual development compared to wild type cells of the same developmental time points (Figure 7A). These results indicate a function of CSN for DenA degradation in *A. nidulans*.

We further investigated whether CSN also affects DEN1 stability in human cells as it had been shown for p27^Kip1^ [47]. DEN1 steady state levels were compared in siGFP and in siCSN1 cells. siGFP cells (control) possess normal levels of CSN and siCSN1 cells are characterized by permanently down regulated CSN amounts [48], [49]. We performed overexpression experiments with transfected constructs of DEN1 (Xpress-DEN1) and CSN1 to study the impact of CSN on DEN1 stability. Ectopically expressed Xpress-DEN1 was much higher in siCSN1 cells as compared to siGFP cells (Figure 7B). Additional overexpression of CSN1 in siCSN1 cells caused a reduction of DEN1 connected with an increase of CSN8 (Figure 7B). We performed inhibitor experiments in order to figure out whether this stability regulation requires proteasomal degradation. Adding the proteasome inhibitor MG132, in addition to cyclohexamide (CHX) treatment, led to a partial accumulation of DEN1 in siGFP cells indicating that DEN1 is degraded at least in part by the proteasome and that the CSN targets it for proteolysis (Figure 7C).

CSN1 is the major DEN1 interacting subunit in human cells, which elevates *de novo* CSN formation [49]. We analyzed whether CSN1 alone can increase DEN1 degradation in HeLa cells. Cells were co-transfected with CSN1wt, with the N-terminal part of CSN1 (1–221), the DEN1 binding fragment, and with the C-terminal fragment of CSN1 (222–527). As shown in Figure 7D overexpression of CSN1wt and of N-terminal CSN1 (1–221) but not of C-terminal CSN1 (222–527) were sufficient to reduce the DEN1 steady state level in HeLa cells significantly. The reduced steady state Xpress-DEN1 level can be restored by MG132 (Figure 7D, right panel) indicating proteasome dependence.

In summary, these data suggest that the COP9 signalosome supports proteasome-dependent protein degradation of DEN1/DenA in fungi and in human cells.

## Discussion

We show here the interplay between the two deneddylases CSN and DEN1/DenA on a molecular and a developmental level of a multicellular organism. CSN and DEN1/DenA physically interact and CSN is involved in DEN1/DenA protein stability regulation. Therefore, CSN is required to control the balance between cellular amounts of the two deneddylases. The physical interaction of the two deneddylases CSN and DEN1 is conserved between different species. There are differences in the affinity of the CSN subunits to DEN1/DenA between organisms. The major DEN1/DenA binding CSN subunit has been changed from CSN2 in *S. pombe* (Figure S4) to CsnG/CSN7 in *A. nidulans* or CSN1 in human cells. This might reflect an evolutionary adaptation to the different complexity of developmental processes in these organisms. Down regulation of CSN results in high steady state concentrations of DEN1 in human cells and fungal cells are unable to reduce DenA protein levels during later phases of asexual development. Elevation of the CSN by overexpressing CSN1 [49] reduced the steady state level of DEN1 in human cells. Since the degradation can be blocked at least in part by MG132, the process is most likely proteasome-dependent (Figure 7D). It has been shown before that the CSN targets p53 for proteasomal degradation *via* phosphorylation by the CSN associated kinase CK2 [50]. In addition, p27^Kip^ is phosphorylated by the CSN which accelerates its degradation by the UPS [51]. At the moment the exact mechanism of CSN mediated proteolysis of DEN1/DenA is not known.

The *Drosophila DEN1* mutation genetically suppresses *Nedd8* mutant lethality [35]. A developmental phenotype of a deletion of the DEN1/DenA encoding gene has not yet been described. The filamentous fungus *A. nidulans* allowed us to dissect the developmental functions of both deneddylases, CSN and DenA/DEN1. Fungal *denA* is crucial for asexual development. In addition, *denA* is required to reduce the sexual program in response to light as an external signal. *A. nidulans* strains deficient in CSN are also unresponsive to light, but are blocked in sexual development and disturbed in secondary metabolism [14], [25], [39], [46]. Defects in *csn* normally do not impair asexual development [14].

Our data suggest that fungal DenA is required to initiate asexual development. After the initial phase of asexual development DenA is normally degraded in a CSN-dependent manner. Defects in *csn* result in stabilized DenA which still allows asexual development. Sexual development and coordinated secondary metabolism require the CSN deneddylase [14], [25], [39].

Deletion of both deneddylase genes generates a strain which accumulates a red pigment indicating that the secondary metabolism of the fungus might be defective. The mutant strain growth predominantly vegetative and is highly impaired in multicellular development. The *denA/csnE* double mutant combines the phenotypes of a Δ*denA* strain which forms only marginal amounts of asexual spores and of Δ*csnE* which is unable to produce sexual fruit bodies. The closest phenotype, reminiscent to the Δ*csnE/ΔdenA* strain, has been recently described for CandA [9], the fungal homolog of human Cullin-associated Nedd8-dissociated protein 1 (CAND1) [52]. In general, CAND1 interaction only occurs to non-neddylated cullins and requires deneddylation as prerequisite [52], [53]. Absence of deneddylation activity could stabilize CRL complexes, thereby altering stability, localization or activity of downstream substrates [54]. Given that CSN5 and DEN1/DenA affect various cullin-dependent and independent targets [35] this might result in developmental phenotypes. The CSN complex might function as a mediator between the two deneddylases and their cognate substrates since it also associates to CRLs [19] and other proteins, such as kinases [20] or USP15 [21]. Just like deneddylation, association of CAND1 is required to facilitate adaptor exchange in cullin complexes like the SCFs. Defects in CAND1 can lead to increased stability of certain types of SCF ligase complexes while the formation of others is abolished [10]. This might explain why, in *A. nidulans*, the defects in sexual differentiation and secondary metabolism of a *csnE* deletion mutant [14], [45], as well as the asexual phenotype of the *denA* deletion strain appear not only in the corresponding double deletion mutant, but also in the fungal *candA* mutants [9]. It is also an indication that both the CSN complex and DenA act in a similar pathway which might converge at the molecular level at the conserved physical interaction of both deneddylases. The CSN affects DenA/DEN1 stability in human and fungal cells. To what extend DenA/DEN1 supports or even substitutes the CsnE/CSN5 mediated deneddylase activity of the CSN complex needs to be resolved. However, only a small fraction of DenA/DEN1 seems to interact with the CSN complex in the living cell as indicated by glycerol gradient experiments from human cells (Figure 6C). Additionally this interaction seems to be very transient suggested from different, repeated pull-down experiments in *A. nidulans* which did not result in co-purification of DenA and CSN subunits with one another (unpublished data).

Our results imply that the DEN1/DenA enzyme is needed for cleaving a specific isopeptide bond between Nedd8 and target protein(s) required for asexual spore formation which cannot be substituted by CSN. Whether this is one of the three *A. nidulans* cullins, even a hyperneddylated cullin or a non-cullin protein needs to be elucidated. Only the double mutant strain shows accumulation of an additional band (Figure 5B) which does not correspond in size to any of the three fungal cullins modified with a single Nedd8 moiety, but would fit with hyperneddylated cullin species. Another possibility are non-cullin target proteins for DEN1 as it had been shown in *Drosophila* where MDM2 is a candidate for DEN1-mediated deneddylation [35]. In mammals DEN1 removes Nedd8 and accelerates MDM2 degradation concomitant with p53 activation [36]. Initially human DEN1 was described as SUMO isopeptidase SENP8 [55] but biochemical *in vitro* experiments showed only marginal activity towards ubiquitin and SUMO substrates [31], [43] whereas preliminary data suggest that a crosstalk between SUMO and Nedd8 might exist in *A. nidulans* (unpublished data).

We conclude that the significance of the CSN-DEN1 interaction consists in regulating the balance between the two deneddylases which have different developmental functions. In more complex organisms the readouts are presumably more complicated. While disruption of CSN is embryonic lethal in plants [28], insects [29] and mammals [15], elevated levels of CSN subunits are connected to certain types of cancer in humans [15], [30]. Furthermore, effector caspases can be neddylated and are thereby inactivated by inhibitors of apoptosis. DEN1 reverses the inactivation by neddylation and stimulates apoptosis [37]. DEN1 is presumably involved in the regulation of apoptosis as an important differentiation program, because protein levels were increased by chemotherapy [36]. These are exciting starting points to elucidate the interplay between CSN and DEN1 in other organisms and to elucidate the role of deneddylases in human tumor formation.

## Materials and Methods

### Cultivation of cells and organisms

Strains of *A. nidulans* used in this study (Table 1) were cultivated on minimal medium [56] and supplements were added as described earlier [57]. Vegetative mycelium was grown in liquid, submerged culture and development was allowed by growth on agar surface at 30°C or 37°C, respectively. Asexual development was induced by enduring white light and formation of sexual fruiting bodies by growth on oxygen limited, tape-sealed plates in the dark [58]. Induction of the nitrate promoter for BiFC was performed on London Medium [1% glucose, 2% salt solution (26 g/L KCl, 26 g/L MgSO_4_, 76 g/L KH_2_PO_4_, 5% (v/v) trace elements) pH 6.5] plus 70 mM NaNO_3_ for induction, or 5 mM NH_4_-tartrate for repression, respectively. Media were supplemented with 100 µM pyridoxine-HCl and/or 5 mM uridine. Selection of transformants carrying the respective resistance cassettes was performed by adding 10 µg/ml phleomycine, 100 ng/ml pyridthiamine or 100 ng/ml nourseothricine (ClonNAT).

**Table 1:** *A. nidulans* strains used in this study.

Name	Genotype	reference
AGB152	*pyrG89;pyroA4*	[45]
TNO2a3	*pyrG89;pyroA4;argB2;*Δ*nkuA::argB*	[71]
AGB209	*pyrG89;pyroA4;*Δ*csnE::pyr4^+^*	[45]
AGB316	*pyrG89;pyroA4;*Δ*denA::pyr4^+^*	this study
AGB318	*pyrG89;pyroA4; *Δ*denA::pyr4^+^;denA^+^;bleo^R^*	this study
AGB457	*pyrG89;pyroA4;argB2;*Δ*nkuA::argB; ^P^nedd8:nTAP:nedd8:pyroA^+^:nedd8^T^*	[27]
AGB461	*pyrG89;pyroA4;*Δ*nkuA::argB;^P^nedd8::pyroA^+^::nTAP::nedd8::nedd8^T^;*Δ*denA::pyr4^+^*	this study
AGB466	*pyrG89;pyroA4;*Δ*nkuA::argB;*Δ*csnE::ptrA^R^*	this study
AGB630	*pyrG89;pyroA4; *Δ*denA::pyr4^+^; niaD^T^::cYFP::niaD^P^/niiA^P^::nYFP::denA::niiA^T^;ptrA^R^*	this study
AGB632	*pyrG89;pyroA4;*Δ*nkuA::argB;*Δ*csnE::ptrA^R^;*Δ*denA::pyr4^+^*	this study
AGB634	*pyrG89;pyroA4;^P^denA::denA::GFP::nat^R^:: denA^T^*	this study
AGB640	*pyrG89;pyroA4;^P^denA:denA::GFP::nat^R^::denA^T^; ^P^gpdA::mRFP::H2A::hisB^T^;pyrG^+^_af_*	this study
AGB641	*pyrG89;pyroA4;mRFP:H2A;phleo^R^*	this study
AGB644	*pyrG89;pyroA4;*Δ*denA:pyr4^+^; niaD^T^::csnG::cYFP::niaD^P^/niiA^P^::nYFP::denA::niiA^T^;ptrA^R^*	this study
AGB708	*pyrG89;pyroA4; ^P^denA::denA::GFP::nat^R^::denA^T^;*Δ*csnG::ptrA^R^, ^P^gpdA::mRFP::H2A::hisB^T^;pyrG^+^_af_*	this study

P = promoter; T = terminator; R = resistance; nat = nourseothricine (clonNAT); ptrA = pyridthiamine; af = *Aspergillus fumigatus*.

*Saccharomyces cerevisiae* transformants were grown on synthetic complex medium [(0.15% yeast nitrogen base without amino acids and (NH_4_)_2_SO_4_, 0.5% (NH_4_)_2_SO_4_, 0.2 mM *myo*-inositol, 0.2% amino acid mix (2 g of each standard-l-amino acid except l-histidine, l-leucine, l-tryptophane plus 2 g l-adenine and 0.2 g p-aminobenzoate] at 30°C. Carbon sources were 2% glucose or 2% galactose/1% raffinose, respectively. Amino acids were supplemented as required.

*Escherichia coli* strain DH5α was employed for the preparation of plasmid DNA and Rosetta or BL21 cells for expression of recombinant *GST::denA*. Bacteria were grown in Luria-Bertani (LB) medium [1% tryptophane, 0.5% yeast extract, 1% NaCl] in the presence of 100 µg/ml ampicillin, and additionally 50 µg/ml chloramphenicol for Rosetta strains. Solid media contained 2% agar.

HeLa cells were grown at standard conditions using RPMI1640 media supplemented by 10% (v/v) FCS and 2 mM glutamine (Biochrom). HeLa siCSN1 cells exhibiting permanently down regulated CSN and mouse B8 fibroblasts stably expressing Flag-tagged CSN2 were generated and cultured as described earlier [18], [48]. For transient overexpression cells were transfected using Lipofectamin LTX (Invitrogen) according to manufacture specifications. Cells were lyzed and examined as outlined previously [49], [50].

### Plasmid construction

Plasmids constructed in this study, as well as primer sequences are given in supporting information Table S1 and Table S2. Details on plasmid construction are described in Text S1.

### *Aspergillus* and yeast strain construction

*Aspergillus nidulans* strains constructed for this study are listed in Table 1. Details on *A. nidulans* strain construction and *S. cerevisiae* strains are given in Text S1.

### Molecular methods

Transformation of *E. coli* and *A. nidulans* was described previously [59], [60]. Isolation of plasmid DNA from *E. coli* was performed using the Qiagen-tip 100 MIDI Kit or Qiagen-tip 20 Plasmid MINI Kit, referring to the producer's manual. Isolation of genomic DNA from *A. nidulans* was carried out as described earlier [61]. *A. nidulans* total RNA was isolated from 0.5 ml of ground mycelia with the Qiagen RNeasy Plant Mini Kit referring to the manufacturer's instructions. Southern hybridization was carried out with non-radioactive probes using the AlkPhos Direct labeling and detection system from GE Healthcare following the manufacturer's guidelines. Northern hybridization was performed according to standard techniques [62]. DNA fragments for hybridization probes, plasmid construction or sequencing were amplified by PCR with the *Taq*- (Fermentas), *Pfu*- (Promega), or Phusion- (Finzymes) polymerase, respectively. *A. nidulans* cDNA was generated from total RNA using the Omniscript RT Kit (Qiagen) following the user's manual. Rapid amplification of cDNA ends (RACE) [41] was achieved by using the GeneRacer Kit (Invitrogen) together with the SuperScriptII reverse transcriptase (Invitrogen) following the protocol provided by the company. DNA sequencing was performed by the Göttingen Genomics Laboratory and Eurofins MWG Operon.

### Quantification methods

Radial growth tests and quantification of conidiospores were described previously [45], [63]. Cleistothecia were quantified from 7 days sexually grown cultures. Surface pictures of plated cultures (150 fold magnification) were collected with an Olympus SZX12 binocular connected to a Kappa PS30 camera. Cleistothecia within a 4×4 field grid of 1 mm^2^ in size were counted and multiplied to obtain the number per cm^2^.

Pixel density values for western quantification were obtained from TIFF files directly generated with the Fusion-SL 4.2 MP detection system (Peqlab) or from digitized X-ray films (Kodak) and analyzed with the ImageJ software (http://rsb.info.nih.gov/ij/index.html). Before comparison, sample density values were adjusted according to an appropriate loading control. The ratio of GFP-DenA and the non-degradable GFP-tag was applied as a measure for steady state DenA stability. No loading control was performed for these experiments as the ratio is independent of the amount of total protein. For all other quantitative experiments means of adjusted density values were compared and observed differences between individual samples were verified by statistical analysis using GraphPad Prism 5.01 (www.graphpad.com) where indicated.

### Computational methods

Blast searches were made at the National Center for Biotechnology Information (www.ncbi.nlm.nih.gov). Sequence analysis was conducted using the Dnastar Lasergene 8.0 software. Protein alignments by ClustalW were carried out at Network Protein Sequence Analysis (http://npsa-pbil.ibcp.fr). Protein motif identification was performed using InterProScan (http://www.ebi.ac.uk/Tools/InterProScan/).

### Protein isolation

Preparation of whole cell extracts from *S. cerevisiae* was described previously [64]. Crude extracts from *Aspergillus* were obtained by grinding mycelia to a fine powder and extraction of soluble proteins with buffer containing 100 mM Tris-HCl, 200 mM NaCl, 20% glycerol, 5 mM EDTA, pH 8 freshly supplemented with Complete protease inhibitor cocktail (Roche), 14 mM ß-mercaptoethanol at 4°C. Protein concentration was estimated using the Bio-Rad assay solution following the manufacturer's guidelines.

### Western experiments

Proteins were denatured in SDS loading dye by heating at 95°C for 10 min and subjected to SDS-PAGE followed by transfer to a nitrocellulose membrane (Whatman). Detection was carried out using the Enhanced ChemiLuminescence (ECL) method described by Tesfaigzi et al. [65], or by using the Pierce detection kit (Thermo Scientific). Signals were recorded on X-ray films (Kodak), Hyperfilm ECL (GE Healthcare) or with a Fusion-SL 4.2 MP detection system (Peqlab).

### Antibodies

Primary antibodies for yeast extracts were directed against Rub1 (N0580-05, US-Biological), Cdc53 (sc-6716, Santa Cruz), LexA (sc-1725, Santa Cruz) and the V5 epitope (R960-25, Invitrogen). GFP fusion proteins were detected using α-GFP antibody (sc-9996, Santa Cruz) and His tagged proteins by α-His-Tag antibody (70796-4, Novagen). For *A. nidulans* experiments α-Actin antibody was purchased from Novus Biochemicals (NB100-74340,) and for experiments with human cells α-Actin and α-γ-Tubulin were purchased from SantaCruz. A Polyclonal antibody directed against Nedd8 was obtained by rabbit immunization with an N-terminal peptide of *A. nidulans* Nedd8 (GenScript). α-DEN1, α-CSN1 and α-CSN8 antibodies were purchased from Enzo, anti-Express from Invitrogen, α-Flag from Stratagene. HRP labeled secondary antibodies were purchased from Jackson Immuno Research, Invitrogen or Seramun.

### Purification of recombinant GST-DenA/DEN1 from *E. coli*

pGEX4T3 (Amersham) plasmids carrying the respective fusion construct were transformed into competent *E. coli* BL21 and transformants selected on LB medium containing ampicillin (100 µg/ml). 10 ml culture was inoculated with a single colony and grown overnight at 37°C on a rotary shaker. 10 ml of overnight-culture were applied to inoculate 250 ml of LB medium. After growth to an OD_600_ = 0.4–0.6 at 37°C on a rotary shaker protein expression was induced by adding 1 mM IPTG and further incubation for 2 h. Cells were harvested by centrifugation resuspended in 10 ml of buffer 1 (20 mM Tris-HCl, 200 mM NaCl, 5 mM DTT) and disintegrated by ultrasonification. The lysate was centrifuged for 15 min at 10.000×g and GST-DenA/DEN1 bound using Glutathione-agarose from Sigma. Beads and supernatant were incubated for 1 h at 4°C with slow rotation. The bead containing solution was transferred to a poly-propylene column (BioRad) and the flow through was discarded. Beads were washed 4 times with 10 ml buffer 1 and proteins were eluted with buffer 2 [50 mM Tris, 200 mM NaCl, 10 mM reduced glutathione pH 8.0]. For further concentration of probes and buffer exchange to PBS pH 7.4, AMICON Ultra filter devices (10 K) were used following the manufacturer's guidelines.

### Biochemical analysis and interaction studies

For glycerol gradient centrifugation cells were lyzed referring to Leppert et al. [49] and density gradient centrifugation was performed in 20 mM Tris; pH 7.2; 50 mM KCl; 1 mM β-mercaptoethanol using a glycerol gradient from 5% to 40% and a rotor TLA 100.3 (Beckman) at 70.000 RPM for 1 h. The gradient was calibrated with purified CSN (about 400 kDa) and with purified 20S proteasome (about 700 kDa). Flag pull downs were described before [18]. Anti-Flag beads and the Flag peptide were purchased from Sigma. Filter-binding assays (far-western experiments) were outlined in detail [66]. The Nedd8-GFP fusion plasmid was kindly provided by Michael Glickman. Protein purification of Nedd8-GFP was carried out under denaturing conditions using Ni-NTA agarose (Qiagen) according to the manufacturer's specifications. Deneddylation of Nedd8-GFP was carried out for 30 min at 37°C in buffer containing 30 mM Tris, 10 mM KCl, 5 mM DTT (pH 7.8). The CUL1 plasmid was obtained from BA Schulman and purified according to [67]. *In vitro* neddylation of the construct consisting of CUL1 associated to RBX1 was performed as described elsewhere [6] and residual unconjugated Nedd8 removed by an additional GST-purification step. Deneddylation was performed in 20 mM Tris HCl, pH 8.0, 200 mM NaCl and 5 mM DTT without prior elution of the Nedd8-CUL1-RBX1 (approximately 60 kDa) from GST-beads. Samples were taken after incubation at 37°C for 30 min, boiled at 95°C for 10 min. in sample buffer and analyzed by western experiments.

### Yeast-2-hybrid

*A. nidulans* protein interactions were tested with the yeast-2-hybrid based interaction trap [68] following existing protocols [14], [69]. *S. pombe* yeast-2-hybrid was performed using the Matchmaker GAL4-based assay (Clontech).

### Microscopy

*A. nidulans* colonies, hyphae and structures were visualized by photography with an Olympus CS30 digital camera combined with an Olympus SZX-ILLB2-200 binocular or a Zeiss Axiolab microscope. Pictures were edited and calibrated for magnification with the cellSens software (Olympus). Fluorescent microscopy was performed using a Zeiss Axio Observer Z.1 system with Zeiss PlanAPOCHROMAT 63×/1,4_oil_ or Zeiss PlanAPOCHROMAT 100×/1,4_oil_ objective, respectively. Pictures were obtained with a QuantEM:512SC (Photometrics) or a Coolsnap HQ^2^ (Photometrics) camera and the SlideBook 5.0 imaging software (Intelligent Imaging Innovations Inc.). Membranes were visualized by staining with 1 µm FM4-64 (Invitrogen). Nuclei were stained with 1 µm DAPI (4′,6-diamidin-2-phenylindol). For localization studies exposure times were GFP: 1500 ms, RFP: 25 ms, DIC: 100 ms and in BiFC experiments exposure times were YFP: 2000 ms, DAPI: 20 ms, DIC: 100 ms.

## Supporting Information

Figure S1 Multiple alignment of the DenA protein deduced from the corresponding gene with related proteins from other organisms. *Aspergillus nidulans* DenA was aligned to sequences of *Aspergillus fumigatus* (XP_749049), *Aspergillus oryzae* (XP_001817262), *Penicillium chrysogenum* (XP_002567908), *Ustilago maydis* (XP_759519), *Schizosaccharomyces pombe* NEP1 (SPBC17D11.01) and NEP2 (SPBC32H8.02c) and *Homo sapiens* (NP_001165582). Sequences of *S. pombe* proteins were N- and C-terminally truncated for the alignment. High consensus residues (>90%) are highlighted in red and low consensus (>50%) in blue. Black arrows indicate the conserved residues (histidine, aspartate, cysteine) forming the catalytic active site of the protein (http://multalin.toulouse.inra.fr/multalin/multalin.html).
(TIF)

Figure S2 Recombinant human DEN1 and *A. nidulans* DenA separated by SDS-PAGE and stained with Coomassie.
(TIF)

Figure S3 Fungal DenA deneddylated human CUL1-Nedd8 substrate *in vitro*. The cleavage reaction increases in parallel with the amount of recombinant DenA added to the reaction mixture. SDS-PAGE and subsequent western experiments show cleavage of the substrate (∼60 kDa) producing the C-terminal CUL1 fragment (∼50 kDa) as outlined in experimental procedures.
(TIF)

Figure S4 Yeast-2-hybrid analysis with *S. pombe* NEP1 and NEP2 and selected CSN subunits. Both isoforms showed a predominant interaction with CSN2 (Bnd = fused to binding domain; Act = fused to activation domain). SNF1 and SNF4 [70] served as control.
(TIF)

Table S1 Plasmids used in this study.
(DOC)

Table S2 Primers used in this study.
(DOC)

Text S1 Supplemental Materials and Methods.
(DOC)
