# Supplementary figures and images for: Control of Multicellular Development by the Physically Interacting Deneddylases DEN1/DenA and COP9 Signalosome
Source: PLoS Genet. 2013 Feb 7;9(2):e1003275. doi: 10.1371/journal.pgen.1003275 (PMC3567183; doi:10.1371/journal.pgen.1003275)

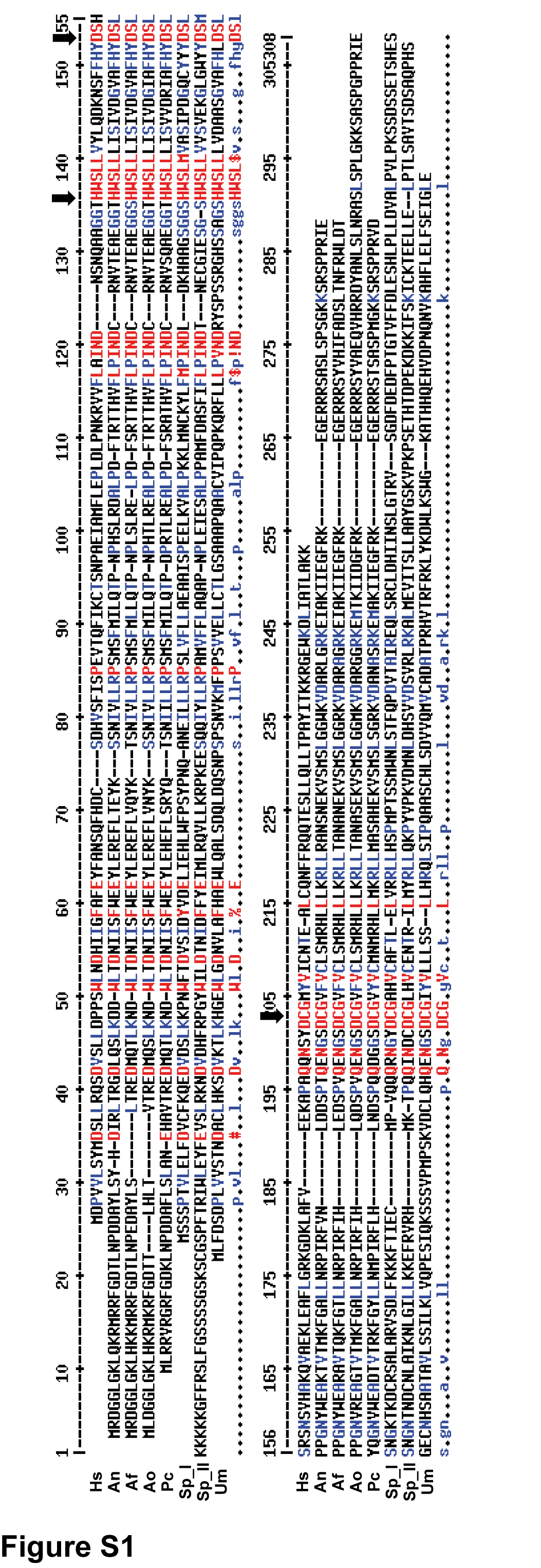

Supplement: Figure S1 — Multiple alignment of the DenA protein deduced from the corresponding gene with related proteins from other organisms. Aspergillus nidulans DenA was aligned to sequences of Aspergillus fumigatus (XP_749049), Aspergillus oryzae (XP_001817262), Penicillium chrysogenum (XP_002567908), Ustilago maydis (XP_759519), Schizosaccharomyces pombe NEP1 (SPBC17D11.01) and NEP2 (SPBC32H8.02c) and Homo sapiens (NP_001165582). Sequences of S. pombe proteins were N- and C-terminally truncated for the alignment. High consensus residues (>90%) are highlighted in red and low consensus (>50%) in blue. Black arrows indicate the conserved residues (histidine, aspartate, cysteine) forming the catalytic active site of the protein (http://multalin.toulouse.inra.fr/multalin/multalin.html). (TIF) [file pgen.1003275.s001.tif]

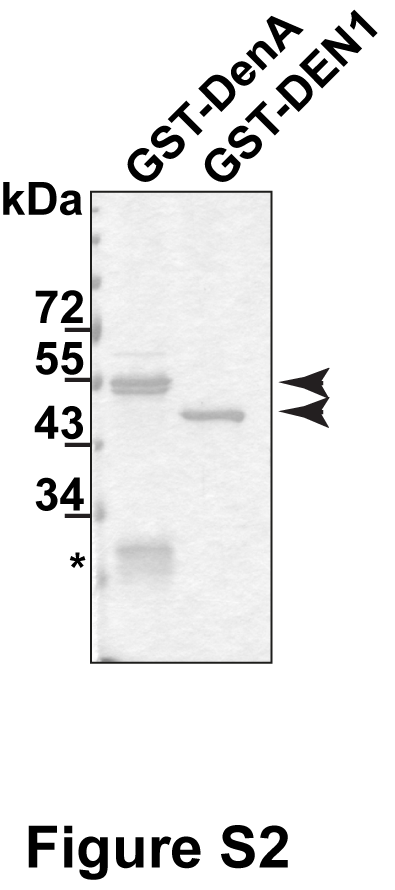

Supplement: Figure S2 — Recombinant human DEN1 and A. nidulans DenA separated by SDS-PAGE and stained with Coomassie. (TIF) [file pgen.1003275.s002.tif]

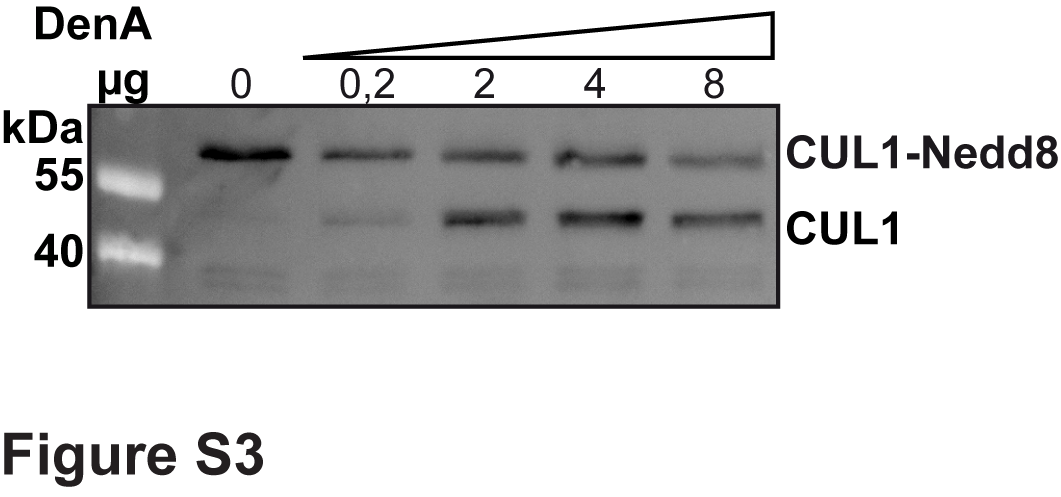

Supplement: Figure S3 — Fungal DenA deneddylated human CUL1-Nedd8 substrate in vitro. The cleavage reaction increases in parallel with the amount of recombinant DenA added to the reaction mixture. SDS-PAGE and subsequent western experiments show cleavage of the substrate (∼60 kDa) producing the C-terminal CUL1 fragment (∼50 kDa) as outlined in experimental procedures. (TIF) [file pgen.1003275.s003.tif]

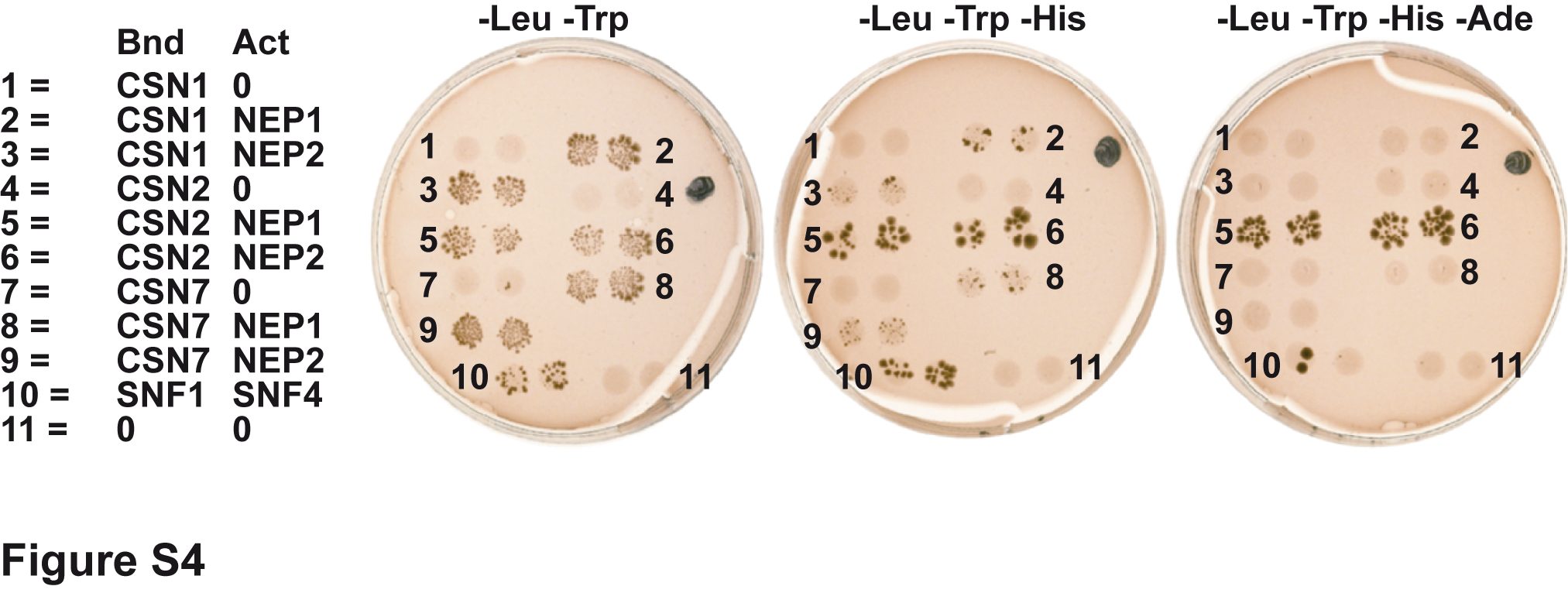

Supplement: Figure S4 — Yeast-2-hybrid analysis with S. pombe NEP1 and NEP2 and selected CSN subunits. Both isoforms showed a predominant interaction with CSN2 (Bnd = fused to binding domain; Act = fused to activation domain). SNF1 and SNF4 [70] served as control. (TIF) [file pgen.1003275.s004.tif]
