# Supplementary material for: Control of Multicellular Development by the Physically Interacting Deneddylases DEN1/DenA and COP9 Signalosome
Source: PLoS Genet. 2013 Feb 7;9(2):e1003275. doi: 10.1371/journal.pgen.1003275 (PMC3567183; doi:10.1371/journal.pgen.1003275)
Supplement: Table S1 — Plasmids used in this study. (DOC) [file pgen.1003275.s005.doc]

**Table S1: Plasmids used in this study**

| **Name** | | **Description** | **Reference** | |
| --- | --- | --- | --- | --- |
| pBluescript®II SK+ | | cloning vector | (Stratagene, La Jolla, CA, USA) | |
| pYES2.1 TOPO-TA | | Yeast expression vector with *GAL1^P^* and C-terminal V5/6xHIS tandem tag | (Invitrogen, Karlsruhe, D) | |
| TOPO-Blunt®II | | cloning vector | (Invitrogen, Karlsruhe, D) | |
| pJET1.2 Blunt | | cloning vector | (Fermentas, St. Leon-Rot, D) | |
| pGEX4-T1 | | *E. coli* expression vector | (GE Healthcare, Freiburg, D) | |
| pcDNA3.1 | | *E. coli* expression vector | (Invitrogen, Karlsruhe, D) | |
| pRG3 | | expression construct for *pyr4* gene from *Neurospora crassa* |  | |
| pEG202 | | yeast-2-hybrid bait vector |  | |
| pJG4-5 | | yeast-2-hybrid prey vector |  | |
| pSK409 | | *^P^niaD*::*niaD^T; P^niiA*::*niiA^T^;ptrA^R^* overexpression vector | S. Krappman pers. communication | |
| pME2357 | | *csnD* cDNA in pJG4-5 |  | |
| pME2501 | | *csnA* cDNA in pJG4-5 |  | |
| pME2978 | | *csnB* cDNA in pJG4-5 |  | |
| pME2979 | | *csnC* cDNA in pJG4-5 |  | |
| pME2980 | | *csnE* cDNA in pJG4-5 |  | |
| pME2981 | | *csnF* cDNA in pJG4-5 |  | |
| pME2982 | | *csnG* cDNA in pJG4-5 |  | |
| pME2983 | | *csnH* cDNA in pJG4-5 |  | |
| pME3267 | | 5'UTR*^denA^*::*denA*::3'UTR*^denA^* in pME3281 | this study | |
| pME3269 | | 3'UTR*^denA^* -PCR-Fragment in TOPO-Blunt®II | this study | |
| pME3270 | | *denA*::5'UTR*^denA^* -PCR-Fragment in TOPO-Blunt®II | this study | |
| pME3271 | | 5'UTR*^denA^* -PCR-Fragment in TOPO-Blunt®II | this study | |
| pME3272 | | *denA*::3'UTR*^denA^* -PCR-Fragment in TOPO-Blunt®II | this study | |
| pME3273 | | *pyr4* -PCR-Fragment in TOPO-Blunt®II | this study | |
| pME3275 | | 5'UTR*^denA^*::*pyr4^+^*::3'UTR*^denA^* in pME3281 | this study | |
| pME3277 | | 5'UTR*^denA^*::*denA*:3'UTR*^denA^* in pME3281 | this study | |
| pME3278 | | *denA* (cDNA) in pYES2.1 in TOPO-TA | this study | |
| pME3279 | | *denA^V5/HIS6^*(cDNA) in pYES2.1 in TOPO-TA | this study | |
| pME3280 | | *culD* (cDNA) in pEG202 |  | |
| pME3281 | | pBluescript®II SK+ with phleomycine resistance cassette |  | |
| pME3674 | | *^P^niaD*::*cYFP*::*rubA^cDNA^*::*niaD^T^; ^P^niiA*::*dcnA^cDNA^*::*nYFP*::*niiA^T^* | this study | |
| pME3857 | | *^P^gpdA::mrfp::H2A::hisB^T^; phleo^R^* in pBlueII SK+ | this study | |
| pME3858 | | *^P^gpdA::mrfp::H2A::hisB^T^; pyrG^af^* in pBlueII SK+ | this study | |
| pME3874 | | *denA* (cDNA) in pEG202 | this study | |
| pME3879 | | *nedd8^precurso^*^r^ (cDNA) in pJG4-5 | this study | |
| pME3881 | | *nedd8^mature^* (cDNA) in pJG4-5 | this study | |
| pME3885 | | *^P^niaD*::*cYFP*::*niaD^T^; ^P^niiA*::*nYFP*::*denA^cDNA^*::*niiA^T^;ptrA^R^* in pSK409 | this study | |
| pME3886 | | *^P^niaD*::*csnG^cDNA^*::*cYFP*::*niaD^T; P^niiA*::*nYFP*::*denA^cDNA^*::*niiA^T^;ptrA^R^* in pSK409 | this study | |
| pME3887 | | *5’UTR^csnG^::ptrA^R^::3’UTR^csnG^* in pJET1.2 | this study | |
| pME3889 | | *denA* (cDNA) in pGEX4-T1 | this study | |
| pME3891 | | 5'UTR*^denA^* -PCR-Fragment 1 [RACE] in pJET1.2 Blunt | this study | |
| pME3892 | | 5'UTR*^denA^* -PCR-Fragment 2 [RACE] in pJET1.2 Blunt | this study | |
| pME3893 | | 5'UTR*^denA^* -PCR-Fragment 3 [RACE] in pJET1.2 Blunt | this study | |
| pME3894 | | 3'UTR*^denA^* -PCR-Fragment 1 [RACE] in pJET1.2 Blunt | this study | |
| pME3895 | | 3'UTR*^denA^* -PCR-Fragment 4 [RACE] in pJET1.2 Blunt | this study | |
| pME3900 | | 5'UTR^denA^::*denA::GFP:nat^R^*::3'UTR*^denA^* in pJET1.2 Blunt | this study | |
| pME3922 | | *denA* (cDNA) in pJET1.2 | this study | |
| pME3924 | | *nedd8^mature^* (cDNA) in pJET1.2 | this study | |
| pME3925 | | *nedd8^precurso^*^r^ (cDNA) in pJET1.2 | this study | |
| pME3929 | | *GFP:nat^R^* cassette in pJET1.2 Blunt | this study | |
|  |  | | |  |

1. Waring RB, May GS, Morris NR (1989) Characterization of an inducible expression system in *Aspergillus nidulans* using *alcA* and *tubulin*-coding genes. Gene 79: 119-130.

2. Golemis E, Brent R (1996) In: Ausubel FM, Brent R, Kingston RE, Moore DD, Seidmann JG et al., editors. Current Protocols in Molecular Biology. New York: Wiley. pp. 429-454.

3. Gyuris J, Golemis E, Chertkov H, Brent R (1993) Cdi1, a human G1 and S phase protein phosphatase that associates with Cdk2. Cell 75: 791-803.

4. Busch S, Schwier EU, Nahlik K, Bayram O, Helmstaedt K, et al. (2007) An eight-subunit COP9 signalosome with an intact JAMM motif is required for fungal fruit body formation. Proceedings of the National Academy of Sciences of the United States of America 104: 8089-8094.

5. Helmstaedt K, Schwier EU, Christmann M, Nahlik K, Westermann M, et al. (2011) Recruitment of the inhibitor Cand1 to the cullin substrate adaptor site mediates interaction to the neddylation site. Mol Biol Cell 22: 153-164.
