# Supplementary material for: Control of Multicellular Development by the Physically Interacting Deneddylases DEN1/DenA and COP9 Signalosome
Source: PLoS Genet. 2013 Feb 7;9(2):e1003275. doi: 10.1371/journal.pgen.1003275 (PMC3567183; doi:10.1371/journal.pgen.1003275)
Supplement: Table S2 — Primers used in this study. (DOC) [file pgen.1003275.s006.doc]

**Table S2:** Primers used in this study

| **Name** | **Sequence (5’🡪3’)** |
| --- | --- |
| MC1 | GTAATCGATGTCATCGCTGAAAAGGG |
| MC2 | CCTGCGGCCGCTCTACATGGGTATGACTAGAG |
| MC3 | GTTGGTCACCGATGGTCTAATCACGAACCTC |
| MC4 | CAAGGTGACCATGCGCGACGGAGGGCTAGG |
| MC5 | GTAGTTAACTATGCGGCATCAGAGCAG |
| MC9 | CCTACTAGTGCGGTATTTCACACCGCATAC |
| MC30 | CAATGCGCGACGGAGGGCTAGG |
| MC31 | TCACTCAATACGCGGCGGACTC |
| MC32 | CTCAATACGCGGCGGACTCC |
| MC71 | AAGAATTCATGCGCGACGGAGGGCTAGG |
| MC72 | TAGAATTCTCACTCAATACGCGGCGGACT |
| MC75 | AGGTCGACATGTTGATCAAGGTCCGTACAC |
| MC76 | GAGTCGACCTACTGAAGGGCGGCGCAGC |
| MC77 | AGGTCGACCTAGCCGCCACGGAGAGCAAGA |
| MC91 | GTCAATTGATGTT ATCAAGGTCCGTACA C |
| MC92 | GTCAATTGCTACTGAAGGGCGGCGCAGC |
| MC93 | GTCAATTGCTAGCCGCCACGGAGAGCAAGA |
| MC94 | TCATGCGCTCCATCGCCACGCGCGACGGAGGGCTAGGAAAGC |
| MC95 | GCTTTCCTAGCCCTCCGTCGCGCGTGGCGATGGAGCGCATGATATAGACGTTGTGGCTG |
| MC96 | ACAGAAGGTCATGAACCACGACCAAGTCCACCATAGGGC |
| MC97 | CTATGACTTCTTCCCAAGAAAACG |
| MC125 | TACCGAGACTATCAAGGGAC |
| MC126 | CATCTAGGCCTCGTGGCTGGTGTTGTTGG |
| MC127 | ACCAGCCACGAGGCCTAGATGGCCTCTTGC |
| MC128 | ACAATGAGATGGGCCACTCAGGCCAATTGA |
| MC129 | CTGAGTGGCCCATCTCATTGTACGGTTCAGG |
| MC130 | TACTCGAGCGCTGCAAAACGAAACACCA |
| MC133 | TCGCCGAATCGCCTCATCCTCTTC |
| MC134 | GTATGAGTTTGGGTGGCTGGAAGGT |
| MC175 | ATCAAGACCCGAGGCAATTTGAC |
| MC176 | CAGGCGCTCTACATGAGCATGCCCTGCCCCTGA  ATAGTTGGCCCGACCGCTTCTAC |
| MC178 | ATCGCCGAATCAGAGGCCAATGT |
| MC179 | GCCCTTGCTCACCATACCACCGCTACCACCCTCAATACGCGGCGGACTCCTC |
| OZG192 | TCAGGGGCAGGGCATGCTCATGTAGAG |
| OZG207 | GGTGGTAGCGGTGGTATGGTGAGC |
| OLKM86 | ATGGCCGACAAGCAGAAGAAC |
| OLKM87 | GTGGTTCATGACCTTCTGTTTCAGGTCGTTCAGGATCTTGCAGGCCGGGCGCTTGTACAGCTCGTCCATG |
| OLKM91 | ATGGTGAGCAAGGGCGAGG |
| DEN1_fw | GCAGAATTCGAGCTCAATGGACCCCGTAGTCTTGAGT |
| DEN1_rv | GCACTCGAGAAGCTTCTACTTTTTAGCAAGTGTGGC |
|  |  |
