# Supplementary material for: Control of Multicellular Development by the Physically Interacting Deneddylases DEN1/DenA and COP9 Signalosome
Source: PLoS Genet. 2013 Feb 7;9(2):e1003275. doi: 10.1371/journal.pgen.1003275 (PMC3567183; doi:10.1371/journal.pgen.1003275)
Supplement: Text S1 — Supplemental Materials and Methods. (DOC) [file pgen.1003275.s007.doc]

**Text S1** Supplemental Materials and Methods

**Plasmid construction**

Gene specific primers MC133 and MC134 together with the primers included in the GeneRacer Kit (Invitrogen) were used to amplify the 5’UTR or the 3’UTR of AN10456, respectively. PCR Fragments obtained from RACE experiments were cloned into pJET1.2 giving pME3891 to pME3895 and subjected to sequencing.

C-terminal fusion of *denA* with *GFP* in plasmid pME3900 was achieved through PCR mediated fusion and ligation of the obtained fragment into the pJET1.2 cloning vector. The *GFP* cassette in combination with a downstream *Nat*-resistance cassette was amplified by PCR with primers OZG207 and OZG192 from plasmid pME3929. PCR on genomic DNA of *A. nidulans* with primers MC178 and MC179 revealed 2402bp combined of the 5’ upstream region and the *denA* ORF with 3’ sequence overhang corresponding to the *GFP* sequence. Amplification with MC175 and MC176 on fungal genomic DNA obtained the 3’ downstream region of *denA* with 5’ sequence overhang for the *Nat*-resistance cassette . All three fragments were used as template for a fusion PCR with primers MC1 and MC2 resulting in the final construct introduced into pJET1.2.Deletion constructs for *A. nidulans* contained the *pyr-4* auxotrophic marker from *Neurospora crassa* amplified from pRG3 by PCR with primers MC5/MC9. The product was inserted into TOPO-BluntII resulting in plasmid pME3273. pME3267 contained the genomic sequence of *denA* with 1200bp flanking region at 5’ and 3’ ends. The plasmid was constructed by PCR amplification of the 5’ flanking region with primers MC1/MC3 and the *denA* open reading frame in addition with the 3’ flanking region by oligonucleotides MC2/MC4 from genomic DNA of *A. nidulans*. PCR fragments were cloned into TOPO-BluntII for the 5’ flanking region to give plasmid pME3271 and into pME3281 via *Not*I/*Eco*RV restriction sites for the ORF in addition with the 3’ flanking region resulting in pME3272, respectively. pME3281 is the pBluescriptII SK+ vector carrying a *phleo* resistance cassette. The 5’ flanking region was excised from pME3271 by cutting with the restriction enzymes *Bst*EII/*Cla*I and ligated into the corresponding restriction sites in pME3272 giving pME3267. Digestion with restriction enzymes *Mfe*I/*Hpa*I removed the *denA* coding sequence from pME3267 but left the flanking regions required for homologous recombination. The *pyr4* marker cassette was excised by digestion with *Eco*RI/*Hpa*I from pME3273 and ligated into the *Mfe*I/*Hpa*I restriction sites of plasmid pME3267 giving plasmid pME3275.

PCR mediated fusion of the *csnG* flanking regions to the *ptrA* resistance cassette was applied to obtain the *csnG* deletion cassette. 1.2 kb 5’ flanking region of *csnG*, containing a downstream overhang for the *ptrA* cassette were amplified with MC125/MC126 from genomic DNA. PCR with primers MC129/MC130 on genomic DNA generated a 2.1 kb fragment of the *csnG* 3’ flanging region with an upstream overhang for *ptrA*. The *ptrA* cassette with overhangs for each *csnG* flanking region was amplified from pSK409 with primers MC127/MC128. All three PCR fragments were assembled in a fusion PCR reaction with primers MC125 and MC130. The deduced fragment was introduced into pJET1.2 resulting in plasmid pME3887.

The *denA* bait plasmid for yeast-2-hybrid interaction experiments was cloned by amplifying the cDNA with primers MC71/MC72 containing *Eco*RI restriction sites. PCR fragments were directly digested by *EcoR*I and ligated into the *Eco*RI sites of pEG202 resulting in plasmid pME3874. Accordingly the *nedd8* cDNAs were amplified with primers MC91/MC92 (*nedd8* precursor form), MC91/MC93 (mature *nedd8*), but with flanking *Mfe*I restriction sites. After digestion with *Mfe*I they were ligated into the *Eco*RI site of pJG4-5 resulting in pME3879 and pME3881, respectively.

Protein fusions with one half of a split *YFP*, for BiFC interaction studies, were obtained by combined fusion PCR and restriction site mediated cloning. The C-terminal part of *YFP* (*cYFP*) was amplified with primers OLKM86 and OLKM87 and the N-terminal *YFP* (*nYFP*) was obtained by primers OLKM91 and MC94 from plasmid pME3674. *csnG* cDNA was amplified from plasmid pME2982 with MC96/MC97 and *denA* cDNA from pME3874 with MC32/MC94. The C-terminal half of *YFP* was fused to the N-terminus of *csnG* in a fusion PCR with primers MC97/OLKM86 and the N-terminal part of *YFP* to the N-terminus of *denA* in a PCR with MC31/OLKM91. Plasmid pME3885 derived from subsequent introduction of the C-terminal half of *YFP* into the *Pme*I site and the fusion of the N-terminal half of *YFP* with *denA* into the *Swa*I site of pSK409, serving as control for unspecific interaction of split *YFP*. pME3886 originates as well from pSK409, subsequently added with *cYFP:csnG* fusion to the *Pme*I site and *nYFP:denA* to the *Swa*I site.

For heterologous expression in yeast the *denA* cDNA was amplified with MC30/MC31 or MC30/MC32 lacking the stop codon at the *denA* C-terminus, respectively. Both fragments were cloned by TA overhangs into pYES2.1 TOPO-TA to give plasmids pME3278 and pME3279. Expression of *denA* was driven by the inducible *GAL1* promoter.

For overexpression and purification of recombinant *denA* the cDNA fragment was excised from plasmid pME3874 with *Eco*RI and ligated into the accordingly linearized pGEX4-T1 resulting in plasmid pME3889.

DEN1 was cloned from a cDNA clone obtained from Imagene and flanked by appropriate restriction sites using the following Primers: DEN1_fw and DEN1_rv. CSN1 and the CSN1 fragments were published before . For transient transfection pcDNA3.1 (Invitrogen) vectors coding for N-terminal Flag- or His-tag were used.

***Aspergillus* and Yeast strain construction**

AGB640 containing a C-terminal fusion of *denA* with *GFP* and the *mrfp::H2A* construct was obtained by transformation of AGB152 with pME3900 resulting in AGB634 and subsequent transformation with pME3858. Homologous recombination of *denA* with the *GFP* fusion construct was proven by Southern hybridization and ectopic integration of the *mrfp::H2A* construct was verified by microscopy. To obtain *csnG* deletion in AGB640 the strain was transformed with the deletion cassette excised from plasmid pME3887 with the restriction enzyme *Xho*I. The cassette mediated resistance against pyridthyamine and was targeted to the *csnG* locus. Successful integration of the deletion construct at the endogenous *csnG* locus was verified by Southern analysis resulting in strain AGB708.

Transformation and selection for uridine/uracil prototrophy was applied to obtain homologous integration of the linear *denA* deletion cassette excised with *Cla*I/*Not*I from pME3275 in strain AGB152 resulting in the *∆denA* strain AGB316. Integration was checked by Southern analysis. Complementation was achieved by ectopic integration of the linearized pME3267 in AGB316 through transformation and selection for the phleomycine resistance marker giving strain AGB318. Transformants were checked by PCR and Southern hybridization.

Strain AGB461 containing a processed variant of Nedd8 (*nedd8m*) at the endogenous locus, combined with the deletion for *denA* was obtained by genetic crossing of strains AGB457 and ABG316 accomplished by Marcia von Zeska Kress. Positive clones were verified by Southern analysis of each locus. The *denA/csnE* double knock-out AGB632 was obtained by transformation of AGB466 with the *denA* deletion cassette excised with *Cla*I/*Not*I from pME3275.

BiFC plasmids pME3885 and pME3886 were transformed into AGB316 resulting in AGB630 and AGB644, respectively. Ectopic integration was verified by Southern analysis. Clones were selected on medium containing phleomycine and lacking uridine and uracil.

For heterologous expression of *A. nidulans* proteins *S. cerevisiae* strain Y03914 and Y06911 (Euroscarf strain collection) were transformed with the plasmids pME3278 and pME3279 and subsequently with pME3280. Wild type strain BY4741 was transformed with plasmid pME3280 as control. Positive transformants were selected on synthetic complex medium [(0.15% yeast nitrogen base without amino acids and (NH_4_)_2_SO_4_, 0.5% (NH_4_)_2_SO_4_, 0.2 mM *myo*-inositol, 0.2% amino acid mix (2 g of each standard-l-amino acid except l-histidine, l‑leucine, l‑tryptophan plus 2 g l-adenine and 0.2 g p-aminobenzoate] lacking histidine and uracil. *A. nidulans denA* and *culD* were expressed in *S. cerevisiae* wild type BY4741 or ∆*rri1/csn5* deletion mutant Y03914 driven by the inducible *GAL1* promoter (*denA*) or the constitutive *ADH1* promoter (*culD*). Resulting DenA was either native or C-terminally fused with a V5/His6 epitope tag. CulD represents an N-terminal fusion with bacterial LexA.

*S. cerevisiae* strains for yeast-2-hybrid tests were obtained in a similar way. Derivatives of the 2-hybrid prey plasmid pJG4-5 containing the cDNA of each *csn* subunit (pME2501, pME2978-79, pME2357, pME2980-83) were used from a previous study . Plasmid pME3922 containing the *denA* cDNA was used as bait. Bait and prey plasmids were subsequently transformed into *S. cerevisiae* EGY48-p1840 and transformants were selected on synthetic complex medium [(0.15% yeast nitrogen base without amino acids and (NH_4_)_2_SO_4_, 0.5% (NH_4_)_2_SO_4_, 0.2 mM *myo*-inositol, 0.2% amino acid mix (2 g of each standard-l-amino acid except l-histidine, l‑leucine, l‑tryptophane, plus 2 g l-adenine and 0.2 g p-aminobenzoate] lacking uracil, histidine and tryptophane.

**Supplemental references**

1. Szewczyk E, Nayak T, Oakley CE, Edgerton H, Xiong Y, et al. (2006) Fusion PCR and gene targeting in *Aspergillus nidulans*. Nat Protoc 1: 3111-3120.

2. Goldstein AL, McCusker JH (1999) Three new dominant drug resistance cassettes for gene disruption in *Saccharomyces cerevisiae*. Yeast 15: 1541-1553.

3. Waring RB, May GS, Morris NR (1989) Characterization of an inducible expression system in *Aspergillus nidulans* using *alcA* and *tubulin*-coding genes. Gene 79: 119-130.

4. Kubodera T, Yamashita N, Nishimura A (2000) Pyrithiamine resistance gene (*ptrA*) of *Aspergillus oryzae*: cloning, characterization and application as a dominant selectable marker for transformation. Biosci Biotechnol Biochem 64: 1416-1421.

5. Busch S, Schwier EU, Nahlik K, Bayram O, Helmstaedt K, et al. (2007) An eight-subunit COP9 signalosome with an intact JAMM motif is required for fungal fruit body formation. Proceedings of the National Academy of Sciences of the United States of America 104: 8089-8094.

6. Huang X, Langelotz C, Hetfeld-Pechoc BK, Schwenk W, Dubiel W (2009) The COP9 signalosome mediates beta-catenin degradation by deneddylation and blocks adenomatous polyposis coli destruction via USP15. Journal of Molecular Biology 391: 691-702.

7. Zhou Z, Wang Y, Cai G, He Q (2012) *Neurospora* COP9 signalosome integrity plays major roles for hyphal growth, conidial development, and circadian function. PLoS Genet 8: e1002712.
